# Individual associations of adolescent alcohol use disorder versus cannabis use disorder symptoms in neural prediction error signaling and the response to novelty

**DOI:** 10.1016/j.dcn.2021.100944

**Published:** 2021-03-15

**Authors:** Joseph Aloi, Kathleen I. Crum, Karina S. Blair, Ru Zhang, Johannah Bashford-Largo, Sahil Bajaj, Amanda Schwartz, Erin Carollo, Soonjo Hwang, Emily Leiker, Francesca M. Filbey, Bruno B. Averbeck, Matthew Dobbertin, R. James R. Blair

**Affiliations:** a Center for Neurobehavioral Research in Children, Boys Town National Research Hospital, Boys Town, NE, United States; b College of Medicine, University of Nebraska Medical Center, Omaha, NE, United States; c Department of Psychiatry, Indiana University School of Medicine, Indianapolis, IN, United States; d Adolescent Behavioral Health Research Program, Indiana University School of Medicine, Indianapolis, IN, United States; e Department of Neuroscience, Medical University of South Carolina, Charleston, SC, United States; f Department of Psychiatry, University of Nebraska Medical Center, Omaha, NE, United States; g Department of Psychiatry, University of Pittsburgh Medical Center, Pittsburgh, PA, United States; h Center for BrainHealth, School of Behavioral and Brain Sciences, University of Texas at Dallas, Dallas, TX, United States; i Section on Learning and Decision Making, Laboratory of Neuropsychology, National Institute of Mental Health, Bethesda, MD, United States

**Keywords:** Adolescent, Alcohol use disorder, Computational modeling, fMRI, Reward prediction error, Striatum

## Abstract

Two of the most commonly used illegal substances by adolescents are alcohol and cannabis. Alcohol use disorder (AUD) and cannabis use disorder (CUD) are associated with poorer decision-making in adolescents. In adolescents, level of AUD symptomatology has been negatively associated with striatal reward responsivity. However, little work has explored the relationship with striatal reward prediction error (RPE) representation and the extent to which any augmentation of RPE by novel stimuli is impacted. One-hundred fifty-one adolescents participated in the Novelty Task while undergoing functional magnetic resonance imaging (fMRI). In this task, participants learn to choose novel or non-novel stimuli to gain monetary reward. Level of AUD symptomatology was negatively associated with both optimal decision-making and BOLD response modulation by RPE within striatum and regions of prefrontal cortex. The neural alterations in RPE representation were particularly pronounced when participants were exploring novel stimuli. Level of CUD symptomatology moderated the relationship between novelty propensity and RPE representation within inferior parietal lobule and dorsomedial prefrontal cortex. These data expand on an emerging literature investigating individual associations of AUD symptomatology levels versus CUD symptomatology levels and RPE representation during reinforcement processing and provide insight on the role of neuro-computational processes underlying reinforcement learning/decision-making in adolescents.

## 1 Introduction

Alcohol use disorder (AUD) and cannabis use disorder (CUD) are among the most common substance use disorders (SUDs) in the United States (Grant et al., 2015; Hasin et al., 2016). Alcohol use and cannabis use during adolescence is associated with an increased risk of developing AUD and/or CUD in adulthood (Winters and Lee, 2008). Individuals with AUD and/or CUD who initiated substance use in adolescence also face a more severe disease course and greater relapse rate (Babor et al., 1992), due in part to weaknesses in decision-making (Kalivas and Volkow, 2005).

Successful decision-making involves the representation of at least two neural computations: (i) expected value (EV) and (ii) reward prediction error (RPE; Rescorla and Wagner, 1972). EV is the subjective value associated with a stimulus and is learned through experience with the stimulus. Responding to a stimulus and gaining reward will result in an increased EV the next time that stimulus is encountered, while responding to a stimulus and gaining punishment will result in a decreased EV the next time that stimulus is encountered. RPE is the difference between the value of the feedback received, and the EV of the stimulus. RPE triggers a revision of EV resulting in more beneficial decisions in the future (Rescorla and Wagner, 1972). Positive/negative RPE is when the feedback value is greater/less than the EV, respectively. Neural regions involved in RPE representation include ventral and dorsal striatum, ventromedial prefrontal cortex (vmPFC), anterior insular cortex (aIC), inferior frontal gyrus (iFG), anterior cingulate cortex (ACC), and posterior cingulate cortex (PCC) (Clithero and Rangel, 2013; O’Doherty et al., 2017).

Adults and adolescents with SUDs show poor performance on decision-making tasks (Gilman et al., 2012; Paulus et al., 2003; Schutter et al., 2011; Whitlow et al., 2004), which may reflect alterations in reinforcement processing (i.e., processes underlying the encoding of positive or negative feedback and/or revising future EV’s following a response to a given stimulus). Work has demonstrated that increased AUD symptoms are associated with reduced striatal responsiveness to non-drug rewards in the context of task-directed paradigms such as the Monetary Incentive Delay (MID) task in adults and adolescents (Aloi et al., 2019; Beck et al., 2009; Claus et al., 2017; Wrase et al., 2007). Less work has examined reinforcement processing in the context of instrumental learning. If long-term substance use reduces reward responsiveness to non-drug rewards, actions associated with non-drug rewards will eventually be extinguished, and these individuals will focus primarily on substance use (and actions associated with substance use) for reward (Heinz et al., 2019; Redish, 2004). Moreover, several treatments for SUDs, such as motivational enhancement and contingency management therapies, rely on instrumental learning (Moos, 2007). As such, if instrumental learning is disrupted in some patients with SUDs, delivery of these treatments will be compromised.

One study with adults reported reduced RPE modulated BOLD responses in SUD patients within the striatum, vmPFC, and aIC (Tanabe et al., 2013). Work with adolescents has reported an inverse relationship between striatal responsiveness and AUD symptoms during feedback on an instrumental learning task (Aloi et al., 2020). Additionally, there has been a report that adolescents with substance abuse histories showed altered RPE representation in PCC in a small sample of substance users (N = 16; White et al., 2016). In short, the current literature on reinforcement processing in the context of instrumental learning in SUDs, particularly its neuro-computational underpinnings, is relatively sparse.

One brain structure that is critical for RPE signaling is the striatum (Clithero and Rangel, 2013; O’Doherty et al., 2017). Furthermore, striatal RPE representation is enhanced in novelty-driven exploration (Costa et al., 2019; Wittmann et al., 2008). Novelty-seeking refers to an organism’s tendency to explore unfamiliar stimuli at the expense of exploiting the EV of familiar stimuli in order to seek out the highest-valued states (Cloninger et al., 1993). Novelty-seeking behavior peaks in adolescence (Kelley et al., 2004) and greater novelty-seeking has been associated with substance use in adolescents (Cinnamon Bidwell et al., 2015). Neuroimaging work has related novelty signaling to an enhancement of striatal signaling during instrumental learning and decision-making when responding to novel stimuli (Wang et al., 2015; Wittmann et al., 2008). Moreover, the striatum is undergoing rapid development during adolescence (Galvan, 2010), and there is evidence that striatal RPE representation in adolescents differs critically from children and adults (Cohen et al., 2010; Nussenbaum and Hartley, 2019). However, no work to date has investigated neuro-computational processes associated with novelty seeking in adolescents with varying levels of AUD/CUD symptomatology.

Adolescents often engage in co-morbid alcohol/cannabis use (Duncan et al., 2015; Moss et al., 2014). While the majority of the literature to date examines AUD and CUD individually (Beck et al., 2009; Jager et al., 2013; Martz et al., 2016; Nees et al., 2015), emerging data indicates that there are differences in individual associations of AUD and CUD symptomatology levels to neuro-cognitive alterations (Aloi et al., 2021, 2020, 2019, 2018; Blair et al., 2019; Leiker et al., 2019). More specifically, prior work has shown that AUD symptomatology level is negatively associated with recruitment of regions implicated in top-down attentional and reward processes (Aloi et al., 2020, 2019, 2018). CUD symptomatology level is negatively associated with recruitment of regions implicated in emotional responding (Aloi et al., 2021; Blair et al., 2019; Leiker et al., 2019). Recent neuropsychological data has suggested that alcohol use is associated with poorer performance on visuospatial tasks while greater cannabis use is associated with poorer performance on inhibitory control and visuospatial tasks (Infante et al., 2020). There are also suggestions that alcohol and cannabis co-use has a significantly greater impact on striatal modulation than use of either substance or no use at all (Claus et al., 2017). In our work, AUD symptomatology level, rather than CUD symptomatology level, has been associated with reduced striatal reward responsiveness (Aloi et al., 2020, 2019). This aligns with other work where the relationship between AUD and reduced reward is more consistently seen (Beck et al., 2009; Nees et al., 2015; Nikolova et al., 2016; Wrase et al., 2007), while the relationship between CUD and striatal reward sensitivity is comparatively more equivocal (Enzi et al., 2015; Filbey et al., 2013; Martz et al., 2016; van Hell et al., 2010). However, no previous work has examined individual associations of AUD versus CUD symptomatology levels and integrity of neuro-computational systems during decision-making and in response to novelty.

The current study aims to address these gaps in the current literature. We made the following predictions: i) On the basis of our previous work (Aloi et al., 2020, 2019), we predicted that AUD, rather than CUD, symptomatology level would be inversely associated with RPE signaling within structures involved in instrumental learning (striatum, vmPFC, dmPFC, ACC, PCC, aIC, iFG); and (ii) AUD, rather than CUD, symptomatology level would be particularly inversely associated with RPE signaling to novel stimuli.

## 2 Methods

### 2.1 Participants

Study participants included 151 adolescents aged 14–18, 111 from a residential youth care facility, and 40 from the surrounding Omaha community. These participants were recruited for part of a broader study investigating neural correlates in adolescents with behavioral and emotional problems. Parental consent and youth assent was obtained from all participants in this study. Twenty-three adolescents were excluded, either due to i) excessive movement during fMRI scanning (*n* = 8; >15 % censored volumes, at >0.5 mm root-mean-squared displacement across adjacent volumes), or ii) scanning/behavioral artifacts (*n* = 15; behavioral artifacts included non-responsiveness on the task, choosing stimuli based on location rather than value). This resulted in a final sample of 128 adolescents; average age = 16.7 (SD = 1.05), average IQ = 98.8 (SD = 11.07), 78 males. Of the final sample of 128 adolescents, 92 were from the residential facility and 36 were from the surrounding Omaha community. For details regarding recruitment, see Supplemental Material.

### 2.2 Measures

#### 2.2.1 SUD assessments

Participants completed both the AUD Identification Test (AUDIT; Fairlie et al., 2006) and the CUD Identification Test (CUDIT; Adamson et al., 2010; Schultz et al., 2019). These scales assess overall symptomatology levels of AUD and CUD over the past year, respectively, and show high validity—as higher scores on these scales are associated with a greater likelihood of an AUD and/or CUD diagnosis, respectively (Adamson et al., 2010; Fairlie et al., 2006; Saunders et al., 1993; Schultz et al., 2019). Cigarette smoking status was determined via the Monitoring the Future Survey (Miech et al., 2016). A Rankit transformation was applied to the AUDIT to reduce skewness/kurtosis. As skewness/kurtosis for the CUDIT were <1, no transformation was applied to the CUDIT. Z-scored, Rankit-transformed AUDIT and z-scored CUDIT were used in all analyses. See Supplemental Material for full results of the Rankit transformation.

#### 2.2.2 Novelty task

**Fig. 1:**
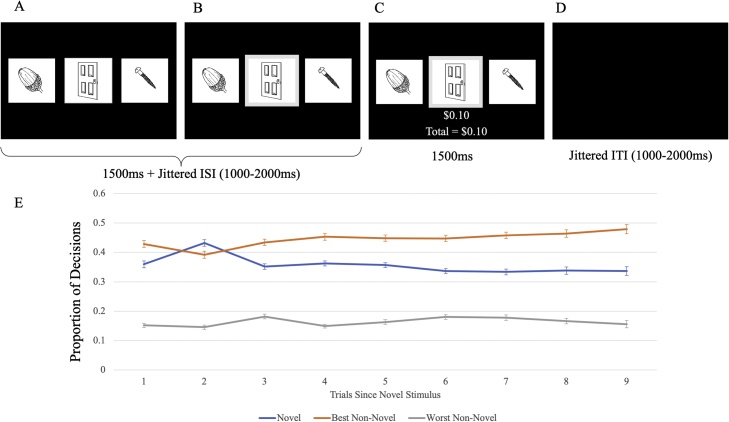
**Diagram of the Novelty Task.** The Novelty Task is a three-armed bandit task where: A) Three stimuli are presented at the beginning of each trial. B) Participant chooses one of the three stimuli. C) Participant receives feedback ($0-$0.30) based on the stimulus chosen on that trial. D) Intertrial interval between trials. E) Proportion of Novel, Best Non-Novel, and Worst Non-Novel stimuli chosen as a function of number of trials since a new novel stimulus is presented. Participants chose the novel stimulus most often on the second trial after a novel stimulus is presented.

The Novelty Task (Fig. 1; Costa et al., 2014; Djamshidian et al., 2011) is a three-armed bandit paradigm where participants are presented with three stimuli on each trial. These stimuli consisted of drawings of everyday objects. Participants are informed prior to beginning the task that each picture has been assigned a unique probability of winning between $0.00–$0.30 and that they would receive 10 % of their overall winnings at the end of the task. Participants won an average of $4.87 over two runs. At the beginning of each trial, the stimuli are presented at one of three randomized locations aligned in a horizontal row. Participants then choose one of the three stimuli via selection on a button box. If the participant responds within 1500 ms, the selected picture is accentuated by a gray border for 1500 ms plus a jittered interval (1000−2000 ms). After this interval, the participant receives outcome feedback indicating their winnings for that trial and their overall winnings. If the participant does not respond, the pictures are replaced by the text “Please respond faster!” After receiving feedback, there is a second jittered interval (1000−2000 ms) before the next choice.

The specific location (left, middle, right) of each stimulus was randomized on each trial. Each stimulus was presented for a series of 5–9 consecutive trials before being replaced with a novel picture. Participants encountered 40 novel stimuli during the course of the task.

### 2.3 Behavioral data

#### 2.3.1 Computational modeling

EV and RPE were modeled using computational modeling techniques developed by Averbeck and colleagues (Averbeck et al., 2013; Costa et al., 2014; Djamshidian et al., 2011). Using this procedure, a reinforcement learning (RL) model was fit to the choice data of participants to determine the following two parameters: learning rate (α) and inverse temperature (β). Based on each participant’s individual behavioral data, a learning curve was modeled establishing EVs and RPEs for each stimulus on each trial. RPE was calculated as the difference between the feedback (F) and the EV for the chosen stimulus with the formula:

$${RPE}_{\left(t\right)}=F_{\left(t\right)}-{EV}_{\left(t\right)}$$

EV was updated for the chosen stimulus for each trial with the following formula:

$${EV}_{\left(t\right)}={EV}_{\left(t-1\right)}+[\alpha \text{*}{RPE}_{\left(t-1\right)}]$$

Therefore, the EV of the current trial (t) equals the EV of the previous trial (t-1) plus the RPE of the previous trial multiplied by the learning rate α. Based on a sample of 290 participants who completed the Novelty Task (including current participants), a learning rate of α = 0.692 was established.

**Fig. 2:**
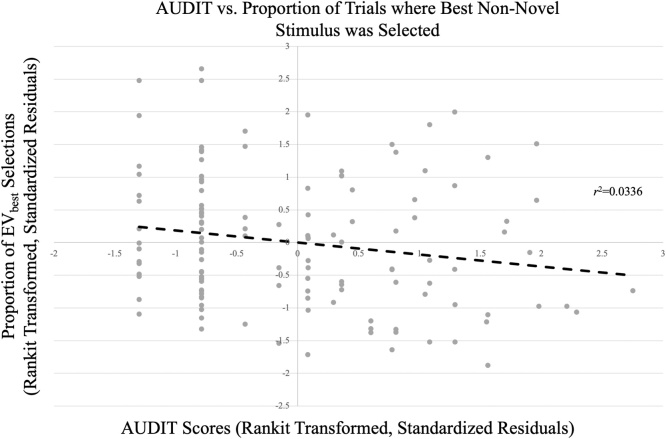
**AUDIT vs. Proportion of Trials Where Highest EV Non-Novel Stimulus was Selected.** Greater AUDIT scores were inversely associated with the proportion of trials where the best non-novel stimulus was selected.

Every 5–9 trials, one of the stimuli were replaced by a novel stimulus. To determine *novelty propensity* (NP), we examined the proportion of times that participants selected the novel stimulus on the *second* trial after the introduction of the novel stimulus. Participants were most likely to pick the novel stimulus on the second trial after its introduction (43.2 % as opposed to 33–36 % on all other trials after introduction; *t*s = 5.77–8.73, *p*s<.001; See Fig. 2). Participants were not more likely to pick the novel stimulus on the first trial after its introduction relative to the third or more trial after introduction (36.0 % as opposed to 33–36 %; *t*s = 0.24–1.87, *p*s>.05). As such, participants were most likely to explore the novel stimulus on the second trial after its introduction; we termed trials where participants chose the novel stimulus on the second trial after its introduction “Explore” trials. All other trials are referred to as “Non-Explore” trials. Briefly, we determined individual novelty propensity (NP) by calculating the EV of the best non-novel stimulus (EV_best_) that predicted a 50 % probability of each participant choosing the novel stimulus on the second trial after its introduction. The average NP across N = 290 adolescents was 0.216, so novel stimuli were assigned an initial EV of 0.216. For details and full results, see Supplemental Material.

### 2.4 Pilot study

To our knowledge, this is the first time the Novelty Task has been used in an adolescent sample. In order to validate the Novelty Task in an adolescent sample and generate hypotheses for this study, we initially piloted the task in a separate sample of 76 typically developing participants without psychopathology or substance use. For further details, including full methods and results, see Supplemental Material.

### 2.5 Functional MRI parameters and analysis

Whole-brain functional MRI data were acquired via a 3 T MAGNETOM Skyra magnetic resonance imaging scanner (Siemens Medical Solutions; see Supplemental Materials for details on MRI parameters). Functional MRI data were preprocessed and analyzed using Analysis of Functional NeuroImages (AFNI) software (Cox, 1996). The first four volumes collected prior to magnetization equilibrium were discarded. The anatomical scan for each participant was registered to the Talairach and Tournoux atlas (Talairach and Tournoux, 1988) and each participant’s functional EPI data were registered to their Talairach anatomical scan in AFNI. Functional images were motion corrected to the initial volume of the first functional run after exclusion of the first four volumes as the reference volume and spatially smoothed with a 6-mm full width at half maximum Gaussian kernel. The data then underwent time series normalization to a T1 image, and these results were multiplied by 100 for each voxel. Therefore, the resultant regression coefficients are representative of a percentage of signal change from the mean.

Data were analyzed with a random-effects general linear model using AFNI. Six task regressors were generated: (1–2) cue phase on non-explore/explore trials, respectively; (3−4) feedback phase on non-explore/explore trials, respectively; and (5–6) stimulus/feedback presentation on trials where participants did not respond, respectively. BOLD response magnitude was parametrically modulated by EV at each voxel/time point and by RPE at each voxel/time point for the cue/feedback phases, respectively. Every volume and its predecessor on which motion exceeded 0.5 mm (Euclidean Norm) was censored. GLM fitting was performed with these six regressors, six motion regressors, and a regressor modeling a baseline drift function. This process generated unmodulated β-coefficients/*t*-statistics for each voxel and regressor. EV-modulated/RPE-modulated β-coefficients/*t*-statistics were generated for the cue/feedback phases on non-explore and explore trials, respectively. The RPE-modulated β-coefficients were used for all fMRI analyses.

### 2.6 Statistical analysis plan

#### 2.6.1 Clinical data

We ran zero-order correlations between AUDIT/CUDIT and (i) psychiatric diagnosis status for the four main co-morbid conditions in our sample: Attention Deficit/Hyperactivity Disorder (ADHD), Conduct Disorder (CD), Major Depressive Disorder (MDD), and Generalized Anxiety Disorder (GAD); (ii) prescribed use of stimulant, antidepressant, or antipsychotic medication; (iii) smoking status; (iv) age; (v) IQ; and (vi) sex. For these analyses, presence of diagnosis/prescribed use of a drug class was coded as 1, absence was coded as 0. The association between AUDIT/CUDIT and smoking status was also determined (smoking scores ranged from 0 to 4; for this analysis, the range of options was coded from 0 (“Never”) to 4 (“Regularly now”)). Steiger’s z-tests were used to compare the relative strength of the relationships between AUDIT versus CUDIT and these variables. Two-sample t-tests were conducted to test for differences between AUDIT/CUDIT and sex. Results are summarized in Table 1.

**Table 1:** Correlations between Demographic and Clinical Variables (N = 128).

	Average (SD)	ADHD^b^ (N = 64)	CD^b^ (N = 62)	MDD^b^ (N = 22)	GAD^b^ (N = 43)	Stimulants^b^ (N = 14)	Antidepressants^b^ (N = 17)	Antipsychotics^b^ (N = 7)	Age	IQ	AUDIT	CUDIT	Smoking
Age	16.7 (1.05)												
IQ	98.8 (11.07)								−0.03				
AUDIT	3.4 (5.48)	0.12	0.37*	0.22†	0.12	−0.05	0.06	0.08	0.07	0.08			
CUDIT	9.1 (9.37)	0.31*	0.47*	0.14	0.17	0.08	0.13	0.02	−0.09	−0.12	0.49*		
Smoking	1.4 (1.51)								0.05	0.02	0.51*	0.56*	
Sex	78 males								0.01^a^	0.07^a^	−0.25*, a	0.06^a^	0.07^a^

ADHD = Attention Deficit/Hyperactivity Disorder, CD = Conduct Disorder, MDD = Major Depressive Disorder, GAD = Generalized Anxiety Disorder, AUDIT = Alcohol Use Disorder Identification Test, CUDIT = Cannabis Use Disorder Identification Test.

† Significant at *p*<0.05.

* Significant at *p*<0.01.

a Correlations coded as 1=male, 0=female.

b Indicates correlation coefficient with variable coded as 1=presence of diagnosis or prescribed substance, 0=absence of diagnosis or prescribed substance.

#### 2.6.2 Computational modeling

To evaluate overall model fit, we ran zero-order correlations between model-predicted proportions of best/worst non-novel stimuli chosen and actual proportions of best/worst non-novel stimuli chosen. We also ran zero-order correlations between AUDIT scores and CUDIT scores and learning rate to ensure that there was no relationship between these measures and learning rate. Finally, we ran linear regressions of actual decision proportions on model-predicted proportions, sex, AUDIT scores, CUDIT scores, AUDIT-by-model prediction interaction, and CUDIT-by-model prediction interaction to determine the consistency of model fit across the distribution of AUDIT and CUDIT scores.

#### 2.6.3 Behavioral correlations with clinical variables

To examine relationships between AUDIT/CUDIT and decision data on the Novelty task, we conducted partial correlations (controlling for sex) between AUDIT/CUDIT and proportion of: (i) best non-novel stimuli chosen on any given trial (i.e., whether the participant’s choice matched the non-novel stimulus associated with the greatest EV); and (ii) NP.

#### 2.6.4 Movement data

Correlational analyses were run to determine potential associations of AUDIT/CUDIT and motion (number of censored volumes, average motion per volume, and maximum displacement).

#### 2.6.5 Functional MRI group analysis

To examine associations between AUDIT/CUDIT scores and RPE representation during Explore vs. Non-Explore trials, a one-way (Decision: Explore, Non-Explore) ANCOVA was run on RPE-modulated BOLD responses using 3dMVM within AFNI (Chen et al., 2014). The following between-subjects variables were included in the model: AUDIT, CUDIT, NP, Sex, AUDIT-by-NP interaction, and CUDIT-by-NP interaction. Sex was included since there was a significant difference between males and females on the AUDIT. Follow-up partial correlations and Steiger’s z-tests were performed within SPSS 26.0 and using freely available online tools (Lee and Preacher, 2013). Follow-up testing of any observed interactions involving more than one covariate were conducted via the PROCESS macro (Hayes, 2013); Johnson-Neyman regions of significance were identified via the PROCESS macro.

#### 2.6.6 Multiple comparison correction

Unless otherwise noted, all clusters were cluster-wise corrected to *p* < .05. Correction for multiple comparisons was performed using a spatial clustering operation in AFNI’s 3dClustSim utilizing the autocorrelation function (-acf) with 10,000 Monte Carlo simulations for the whole-brain analysis. Spatial autocorrelation was estimated from residuals from the individual-level GLMs. In line with current recommendations, the initial threshold was set at *p* = .001 (Cox et al., 2017). This process yielded an extent threshold of *k* = 17 contiguous voxels for the whole brain (NN1/facewise neighbor clustering). We also report clusters within striatum/medial prefrontal cortex (mPFC) that are significant at a more lenient extent threshold of *k* = 10 contiguous voxels, given the importance of these regions in RPE representation. Follow-up analyses were conducted on the percent signal change taken from all significant voxels within each functional ROI generated by AFNI to examine significant main effects and interactions with planned follow-up testing within SPSS 26.0. Effect sizes for all clusters are reported in order to facilitate meta-analyses; although it should be noted that effect sizes in fMRI datasets can be inflated (Geuter et al., 2018; Reddan et al., 2017; Yarkoni, 2009).

## 3 Results

### 3.1 Clinical data

Of the final sample of 128 adolescents, 96 adolescents endorsed past-year use of either alcohol and/or cannabis. All adolescents in the residential facility had been abstinent from any substance use for at least 4 weeks prior to scanning. AUDIT ranged from 0 to 34 [*M* = 3.4, *SD* = 5.48] and CUDIT ranged from 0 to 32 [*M* = 9.1, *SD* = 9.37]. With regard to past-year quantity/frequency reported on the AUDIT, the average score for the quantity item was 0.8 [*SD* = 1.19; approximately 3−4 drinks per alcohol use occasion] and the average score on the frequency item was 0.9 [*SD* = 1.01; approximately once per month]. With regard to past-year quantity/frequency reported on the CUDIT the average score for the quantity item was 1.9 [*SD* = 1.73; approximately 3−4 h “stoned” per cannabis use occasion] and the average score for the frequency item was 1.6 [*SD* = 1.31; approximately 2–4 times per month]. Seventy-six adolescents met the clinical cutoffs on the AUDIT and/or CUDIT suggestive of adolescent AUD (AUDIT ≥ 4) or CUD (CUDIT ≥ 6; Fairlie et al., 2006; Schultz et al., 2019). Forty participants had an AUDIT ≥ 4 and 67 participants had a CUDIT ≥ 6. Consistent with prior work indicating high rates of poly-substance use in adolescents (Mason et al., 2013), 31 participants had both an AUDIT ≥ 4 and CUDIT ≥ 6. Of the 76 participants who met the clinical cutoffs on the AUDIT and/or CUDIT, 72 were from the residential facility and 4 were from the community.

Correlation analyses revealed a strong positive relationship between AUDIT and CUDIT scores [*r* = 0.49, *p* < .001]; see Table 1 (though note Variance Inflation Factors [VIFs] for AUDIT and CUDIT were 1.49 and 1.42 respectively indicating that multicollinearity was not a significant concern). In all cases with the exception of ADHD, follow up Steiger’s z tests showed no significant differences in the strengths of association between AUDIT/CUDIT scores and psychiatric diagnoses [Steiger’s Z’s=−1.20 to 0.94, *p*’s>.05]. CUDIT scores were significantly more associated with ADHD symptoms than AUDIT scores [Steiger’s Z = 2.13, *p* < .05]. Additionally, while AUDIT and CUDIT scores were both associated with smoking [*r*’s = 0.51–0.56, *p*’s<.05], there were no significant differences in the strengths of association between AUDIT/CUDIT scores and smoking [Steiger’s Z = 0.69, *p* > .05].

There were no significant relationships between AUDIT/CUDIT scores and IQ [*r*’s=−.12 to .08, *p*’s>.05] or AUDIT/CUDIT scores and age [*r*’s=−.09 to .07, *p*’s>.05]. However, a two-sample *t*-test revealed that females showed greater AUDIT scores than males [*t*(126) = 2.84, *p* < .01]. The average AUDIT score for females was 5.1 [SD=7.20] and the average AUDIT score for males was 2.4 [SD=3.70]. There was no association between CUDIT scores and sex. The average CUDIT score for females was 8.3 [SD=9.73] and the average CUDIT score for males was 9.6 [SD=9.16].

### 3.2 Behavioral data

#### 3.2.1 Behavioral correlations with clinical variables

Partial correlations (controlling for sex) were run between AUDIT, CUDIT, proportion of best non-novel stimuli chosen, and NP. There was a negative association between AUDIT and proportion of best non-novel stimuli chosen [*r*(125)=−0.18, *p* < .05; Fig. 2]. However, there was no association between AUDIT and NP [*r*(125) = .05, *p* > .05]. There was no association between CUDIT and both proportion of best non-novel stimuli chosen [*r*(125)=−0.08, *p* > .05] and NP [*r*(125)= −.02, *p* > .05].

### 3.3 fMRI results

#### 3.3.1 Movement data

There were no significant correlations between AUDIT scores or CUDIT scores and number of censored volumes, average motion per volume, or maximum displacement [*r*s=-.02−.15, *p*s>.05].

### 3.4 BOLD response data

Our ANCOVA revealed regions showing a main effect of AUDIT as well as regions showing AUDIT-by-Explore, CUDIT-by-NP and CUDIT-by-NP-by-Explore Interactions. No regons showed significant AUDIT-by-NP, AUDIT-by-NP-by-Explore or CUDIT-by-Explore interactions.

#### 3.4.1 Main effect of AUDIT

**Fig. 3:**
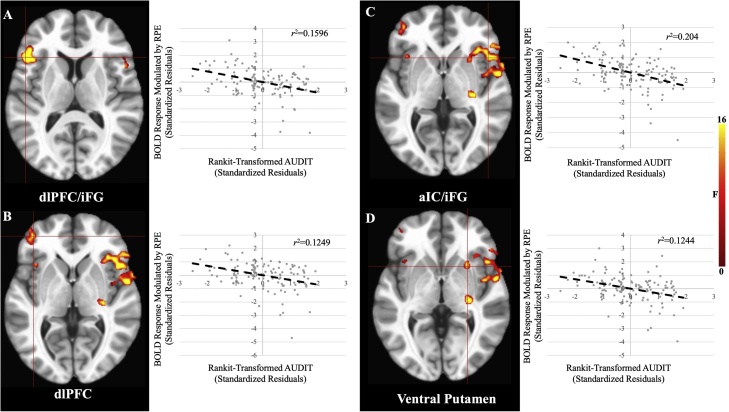
**Main Effects of AUDIT** within A) dlPFC/iFG, B) dlPFC, C) aIC/iFG, and D) Ventral Putamen. In all cases, greater AUDIT scores were associated with reduced BOLD response modulation by RPE. Note: the listed  *r*^2^ values are post-hoc tests and are for the purposes of visualization and interpretation only.

There was a significant main effect of AUDIT within bilateral aIC, iFG, dlPFC, inferior parietal lobule (iPL), and, at a lenient extent threshold (*k*>10 voxels), ventral putamen (Fig. 3, Table 2). In these brain regions greater AUDIT scores were associated with reduced modulation of BOLD response by RPE. Three additional regions outside of striatum/mPFC were significant at the lenient extent threshold: dlPFC, precentral gyrus, and thalamus.

**Table 2:** Brain regions demonstrating significant AUDIT, AUDIT-by-Explore, CUDIT-by-NP, and CUDIT-by-NP-by-Explore effects.

Coordinates of Peak Activation^b^								
Region^a^	Hemisphere	BA	x	y	z	*F*	Partial η^2^	Voxels
Main Effect of AUDIT								
Ventral Putamen^c^	R	–	26	14	−1	17.20	0.124	11
dlPFC	R	9/10	29	50	26	17.55	0.127	26
dlPFC	L	10/46	−37	44	5	17.27	0.125	24
dlPFC	L	9/10	−22	44	26	16.17	0.118	16
aIC/iFG	R	22/45/ 47/13	53	−1	2	31.02	0.204	117
aIC/iFG	L	32	−46	20	11	22.99	0.160	48
iFG	R	11	38	35	−10	24.85	0.170	21
Precentral Gyrus^c^	L	6	−55	2	29	20.69	0.146	13
Supramarginal Gyrus/iPL	L	40	−49	−49	32	17.10	0.124	23
Cerebellum	R	–	32	−52	−37	25.29	0.173	19
Cerebellum	L	–	−31	−49	−28	26.92	0.182	38
Thalamus^c^	R	–	29	−19	2	19.19	0.137	15
AUDIT-by-Explore								
Caudate	L	–	−16	5	14	16.08	0.117	20
Ventral Putamen/aIC/iFG	R	47	47	17	2	25.17	0.172	79
aIC/iFG	L	45	−46	20	11	18.29	0.131	23
dlPFC	L	10/46	−37	44	5	18.43	0.132	26
ACC^c^	L	32	−10	35	17	16.29	0.119	13
MFG	R	47	35	32	−4	20.71	0.146	21
iPL^c^	L	40	−49	−52	35	15.42	0.110	16
CUDIT-by-NP								
iPL	R	40	47	−40	38	21.04	0.148	39
Cerebellum	R/L	–	5	−76	−28	19.78	0.141	19
CUDIT-by-NP-by-Explore								
dmPFC	R/L	32	2	20	41	14.73	0.109	21
iPL	R	40	47	−40	38	20.46	0.145	31
Superior Temporal Gyrus	R	13	59	−43	17	16.53	0.120	25

Note:

a According to the Talairach Daemon Atlas (http://www.nitrc.org/projects/tal-daemon/).

b Based on the Tournoux & Talairach standard brain template.

c Below the ClustSim established threshold, BA = Brodmann’s Area.

#### 3.4.2 AUDIT-by-explore interaction

**Fig. 4:**
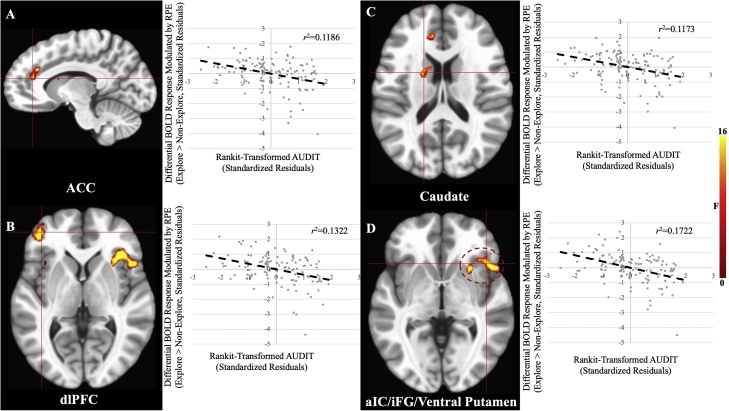
**AUDIT-by-Explore Interaction Effects** within A) ACC, B) dlPFC, C) Caudate, and D) aIC/iFG/Ventral Putamen. In all cases, greater AUDIT scores were associated with reduced BOLD response modulation by RPE for explore relative to non-explore trials. Note: the listed  *r*^2^ values are post-hoc tests and are for the purposes of visualization and interpretation only.

There was a significant AUDIT-by-Explore interaction within caudate, ventral putamen, aIC, iFG, dlPFC, iPL, and at a lenient extent threshold, ACC (Fig. 4, Table 2). In all brain regions greater AUDIT scores were associated with reduced modulation of BOLD response by RPE in explore relative to non-explore trials. One additional region outside of striatum/mPFC was significant at the lenient extent threshold: iPL.

#### 3.4.3 CUDIT-by-NP interaction

There was a significant CUDIT-by-NP interaction within iPL and cerebellum. In both brain regions, NP was positively associated with RPE modulated BOLD responses in individuals with low CUDIT scores (<4 [Cerebellum], <6 [iPL]); NP was negatively associated with RPE modulated BOLD responses in individuals with high CUDIT scores (>13 [Cerebellum], >15 [iPL]).

#### 3.4.4 CUDIT-by-NP-by-explore interaction

**Fig. 5:**
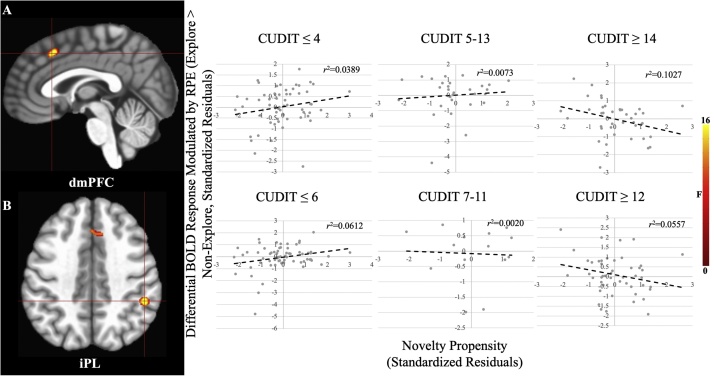
**CUDIT-by-NP-by-Explore Interaction Effects** within A) dmPFC and B) iPL. In both cases, for individuals with low CUDIT scores (≤4 [dmPFC], ≤6 [iPL]) there was a positive relationship between NP and RPE modulation for explore relative to non-explore trials. However, in individuals with high CUDIT scores (≥14 [dmPFC], ≥12 [iPL]) there was an inverse relationship between NP and RPE modulation for explore relative to non-explore trials. Note: the listed  *r*^2^ values are post-hoc tests and are for the purposes of visualization and interpretation only.

There was a significant CUDIT-by-NP-by-Explore interaction within dmPFC, iPL, and superior temporal gyrus (STG) (Fig. 5, Table 2). In these regions, NP was positively associated with RPE modulated BOLD responses in individuals with low CUDIT scores (<4 [dmPFC & STG], <6 [iPL]); NP was negatively associated with RPE modulated BOLD responses in individuals with high CUDIT scores (>12 [dmPFC and STG], >14 [iPL]).

### 3.5 Potential confounds

The current sample has several potential confounds, including co-morbid psychiatric concerns, psychotropic medication usage, smoking/tobacco use, suppression effects, community participants with clinically significant AUDIT/CUDIT scores, and age. Briefly, we conducted an additional analysis for each potential confound that repeated the main analysis controlling for that specific confound. Each of these analyses yielded results similar to the main analysis (for fuller descriptions, see Supplemental Material).

## 4 Discussion

The first goal of the current study was to investigate the extent to which AUD/CUD symptomatology level was associated with RPE signaling. In line with our hypotheses, greater AUDIT scores were associated with reduced modulation by RPE within ventral putamen (at a lenient extent threshold) and attentional regions such as dlPFC, iFG, aIC, and iPL, as well as with poorer performance on the task (i.e., proportion of best non-novel stimuli chosen). The second goal of the current study was to investigate the extent to which AUD/CUD symptomatology level was associated with RPE signaling to novel stimuli. We also found that during novel stimulus exploration, greater AUDIT scores were associated with diminished RPE modulation within ventral putamen, caudate, dlPFC, iFG, aIC, iPL, and at a lenient extent threshold, ACC. Finally, we found that NP was positively associated with RPE modulation within attentional regions such as iPL, dmPFC, and STG in individuals with subclinical CUDIT scores (0–6), but not in individuals with clinically significant CUDIT scores.

We predicted that AUD symptomatology level would be negatively associated with RPE signaling within striatum. This was because of previous work indicating that chronic substance use leads to hypo-responsiveness to non-drug reward related cues within the striatum in non-learning (Aloi et al., 2020, 2019; Beck et al., 2009; Tapert et al., 2001; Wrase et al., 2007) and instrumental learning (Aloi et al., 2020) paradigms. In line with this hypothesis, we found that greater AUD symptomatology level was associated with reduced RPE modulation within ventral putamen (at a lenient extent threshold). Most previous work looking at reward sensitivity in individuals with AUD has used the MID task (Aloi et al., 2019; Beck et al., 2009; Nees et al., 2015; Wrase et al., 2007). This task examines response to reward outcomes where reinforcement contingencies are determined by the task structure and known to the participants. Little work has examined reward responsiveness in the context of instrumental learning and, even so, analyses have not adopted a neuro-computational approach (Aloi et al., 2020). The current data extend the previous literature by showing that greater AUD symptomatology level is associated with reduced *RPE representation* within striatum. On the basis of these data, AUD symptomatology level is negatively associated with computations signaling the unexpectedness of received (non-drug) reward. Thus, instrumental learning and choosing actions that engender non-drug rewards should be compromised.

The current data indicate similar effects with regard to attentional regions, such as dlPFC, iPL, aIC, and iFG. Dorsolateral PFC and iPL comprise a central executive network that is associated with working memory and decision-making (Katsuki and Constantinidis, 2012), while aIC is implicated in salience detection and learning from uncertain rewards (Preuschoff et al., 2008). However, caution is warranted regarding reverse inference as the current study is unable to delineate the precise roles of these brain regions with regard to attentional subsystems in the Novelty Task. Prior literature has shown that AUD symptomatology level is associated with alterations within dlPFC, iPL, aIC, and iFG during tasks designed to elicit attentional neuro-circuitries (Ahmadi et al., 2013; Aloi et al., 2018; Thayer et al., 2015). However, previous work from our group has shown that in reward paradigms, reduced striatal responsivity in adolescents with greater AUD symptomatology level is also associated with reduced attentional responsiveness (Aloi et al., 2020, 2019). Moreover, prior work in adults has shown that alterations in dlPFC-striatal connectivity during an instrumental learning task in individuals with AUD predicted poorer performance on the task (Park et al., 2011). We hypothesize that the current data represent an alteration in orchestrating an attentional response to RPE.

Reduced RPE signaling should manifest behaviorally as poorer performance in reinforcement-based decision-making (Schultz, 2016). In line with our neuroimaging findings that AUDIT scores were negatively associated with RPE sensitivity, AUDIT scores were also negatively associated with behavioral performance on the current task. This aligns with previous work showing that individuals with AUD show poor performance in reinforcement-based decision-making tasks (Bechara et al., 2001; Tomassini et al., 2012) – though see (Aloi et al., 2020). Importantly, understanding alterations in neuro-computational processes in reward learning associated with AUD has significant clinical implications (García-García et al., 2017; Schultz, 2016). Reduced RPE responsiveness to non-drug reinforcers will result in these being less reinforcing, thus reducing the individual’s propensity to engage in actions that might engender these reinforcers—particularly relative to those associated with drug reinforcers where signaling may be increased (García-García et al., 2017; Schultz, 2016). Contingency management techniques based on principles of reinforcement learning are effective in adults with SUDs (Petry and Simcic, 2002). However, more thorough understanding of the mechanisms underlying these interventions *in adolescents* is needed given the maturation of neurobiological systems underlying reinforcement learning during this time period (Galvan, 2010; Nussenbaum and Hartley, 2019). Potentially, if interventions do not address these reinforcement-related difficulties, it will be difficult for these individuals to shift behavioral/attentional foci away from drug-related actions and towards other reward-related actions.

As noted above, striatum shows an augmented response to novel reinforcements in addition to its role in responding to RPE, as striatal RPE representation is enhanced during novelty-driven exploration (Costa et al., 2019; Wittmann et al., 2008). This function of striatum was also disrupted as a function of AUD symptomatology level; AUDIT scores were negatively associated with extent of increase in ventral putamen and caudate RPE modulation when exploring novel stimuli. This finding could be considered surprising since prior work has indicated that individuals with i*ncreased* sensitivity to novel stimuli are particularly at risk for developing substance use behaviors (Cinnamon Bidwell et al., 2015; Ray et al., 2009). However, it is important to note the distinction between *neuro-developmental risk factors for initiating substance use* as opposed to the *neuro-developmental impacts of substance use*. Increased reward responsiveness and *increased* novelty seeking are risk factors for the *development* of substance use (Cinnamon Bidwell et al., 2015; Heitzeg et al., 2015; Hulvershorn et al., 2015; Ray et al., 2009). Yet, the current data, and considerable previous work (Aloi et al., 2020, 2019; Nees et al., 2015), indicate that AUD symptomatology level is associated with *decreased* reward responsiveness and, in the current study, reduced RPE signaling to novel stimuli. In short, there appear to be marked differences, at least with respect to non-drug reward sensitivity/RPE representation, in the *neurobiological risk factors for substance use* relative to the *neurobiological consequences of severe substance (alcohol) use*.

In initial analyses with healthy participants, we observed that the augmentation of the RPE signal to novel stimuli was particularly marked in individuals with greater NP (Fig. S2). In the current study, this was seen individuals with sub-clinical CUDIT scores within iPL, dmPFC, and STG. However, in individuals with high CUD symptomatology levels, there was reduced differential RPE modulation to novel vs. non-novel stimuli as a function of NP. This suggests that increased CUD symptomatology level is associated with a degree of compromised responsiveness to novelty, at least within regions implicated in attentional responding. Notably, previous work has shown that individuals with greater CUD symptomatology levels show reduced ACC/dmPFC responsiveness and awareness to error feedback (Aloi et al., 2019; Hester et al., 2009), which may be associated with “amotivational syndrome”, often observed in CUD (Pacheco-Colon et al., 2018). These data may indicate particular toxic impacts of cannabis use.

Prior work has suggested that individuals with SUDs show reduced striatal responsiveness to non-drug cues because chronic substance use results in dopamine receptor downregulation and reduced dopaminergic neurotransmission within this structure (Koob and Volkow, 2016). It has been suggested that chronic alcohol use and chronic cannabis use reduce synaptic dopamine to non-alcohol and non-cannabis stimuli, respectively (Koob, 2013; Martinez et al., 2005; Van De Giessen et al., 2017). Notably, however, molecular data indicates individual associations of neuronal impact for long-term alcohol and cannabis use. While both acute alcohol use and acute cannabis use induce increases in synaptic dopamine in the ventral striatum, the increase following alcohol appears larger (Boileau et al., 2003; Bossong et al., 2009). Moreover, individuals with AUD show reduced striatal dopamine receptor availability (Martinez et al., 2005; Trifilieff and Martinez, 2014) while individuals with CUD do not (Stokes et al., 2012; Trifilieff and Martinez, 2014). We believe our individual associations of AUDIT versus CUDIT scores with RPE may reflect these molecular effects; we speculate that prolonged alcohol use exerts a greater impact on striatal and cortical dopamine receptors long-term, leading to broader alterations in RPE representation relative to prolonged cannabis use.

The current study has several limitations. First, urine/breathalyzer testing for alcohol or cannabis use was not conducted at the time of scanning. However, all but two participants with significant alcohol and/or cannabis use histories were residents of a highly supervised residential youth care facility, and subject to random drug testing as part of the treatment program for at least four weeks prior to scanning. Exclusion of these two participants elicited highly similar results. Second, the present study was cross-sectional, so the results reported might reflect the effects of AUD/CUD on the developing brain or pre-existing risk factors for AUD/CUD. However, the current results reflect individual associations of AUD/CUD symptomatology level and RPE signaling; it is unclear that there are pre-existing neural risk factors that place individuals specifically at risk for AUD rather than CUD (or vice-versa). Third, there was a great degree of psychiatric co-morbidity within the sample. It could be argued that the findings in the current study are reflective of psychiatric co-morbidities of AUD/CUD rather than AUD/CUD itself. Notably, our supplemental analyses showed that including ADHD, CD, MDD, or GAD diagnoses as covariates did not significantly alter the main results. Additionally, a number of participants were prescribed psychotropic medications for these conditions. While this use might have influenced our findings, it is important to note that including stimulant, antidepressant, or antipsychotic use as covariates did not significantly alter the main results (see Supplementary Tables S7–S9). Therefore, the current findings likely reflect level of AUD symptomatology rather than any psychiatric co-morbidity or psychotropic medication use. Relatedly, AUD and CUD were highly co-morbid in our sample; of the 40 adolescents with AUDIT ≥ 4, only 9 did not have CUDIT ≥ 6. Such findings are common in adolescents; epidemiological data indicate that co-morbid alcohol/cannabis use is common (Mason et al., 2013). This makes interpretation of group-based studies very complex unless a clinically atypical single drug use group is identified. However, our dimensional approach enables identification of individual associations related to AUD versus CUD symptomatology levels. Nevertheless, future longitudinal work in groups of single drug use individuals will be necessary to determine causal effects of alcohol use only versus cannabis use only. Similarly, AUDIT and CUDIT scores were correlated, and so inclusion of both AUDIT and CUDIT scores could confound one another due to multicollinearity. However, the VIFs of the AUDIT and CUDIT regressors in our model were 1.49 and 1.42, respectively, suggesting that this was not the case. It should also be noted AUDIT and CUDIT are general indices of AUD and CUD symptomatology levels, respectively. These measures index rough levels of quantity/frequency, levels of abuse, and levels of dependence of alcohol and cannabis over the past year. However, additional indices such as age of first use and overall length of substance use, were unavailable for this sample. Future work is necessary to delineate the association between these important substance use parameters and RPE signaling. Finally, it should be noted that the Novelty task utilized monetary reward. A subject of future investigation should be whether these relationships between AUD symptomatology level and RPE signaling are consistent with regard to primary rewards (e.g., food). Relatedly, future work should also investigate the relationship between SUDs and RPE signaling with regard to substance-related stimuli. In particular, this work should focus on the relationship between AUD and RPE signaling with regard to alcohol-related stimuli.

In summary, AUDIT scores were inversely related to RPE signaling within ventral putamen, iFG, dlPFC, and iPL. We also found that in individuals with greater AUDIT scores, RPE signaling within caudate, ventral putamen, dlPFC, iFG, iPL, and ACC was particularly reduced when exploring novel stimuli. Furthermore, our data suggest that greater AUDIT scores were associated with weaker task performance. Regarding CUD symptoms, we found a positive relationship between NP and RPE modulation within dmPFC and iPL only in individuals with sub-clinical CUDIT scores. These data replicate prior work (Aloi et al., 2020, 2019) indicating that AUD symptomatology level is associated with alterations in striatal/cortical systems when receiving rewards and extend this finding by establishing that (i) AUD symptomatology level is associated with altered RPE signaling and (ii) this alteration is particularly pronounced when exploring novel stimuli.

## Data availability statement

The data that support the findings of this study are available from the corresponding author upon reasonable request. The data are not publicly available due to IRB restrictions.

## Declaration of Competing Interest

The authors report no declarations of interest.
