# Supplementary material for: Individual associations of adolescent alcohol use disorder versus cannabis use disorder symptoms in neural prediction error signaling and the response to novelty
Source: Dev Cogn Neurosci. 2021 Mar 15;48:100944. doi: 10.1016/j.dcn.2021.100944 (PMC8024914; doi:10.1016/j.dcn.2021.100944)
Supplement: Supplementary file 1 [file mmc1.docx]

Table of Contents

[*Pilot Study* 1](#_Toc62332066)

[*Introduction* 1](#_Toc62332067)

[*Methods* 1](#_Toc62332068)

[*Results* 2](#_Toc62332069)

[*Discussion* 2](#_Toc62332070)

[Main Study: Supplemental Methods 3](#_Toc62332071)

[*Recruitment* 3](#_Toc62332072)

[*Computational Modeling: Reinforcement Learning Model* 4](#_Toc62332073)

[*Computational Modeling: Novelty Propensity* 5](#_Toc62332074)

[Main Study: Supplemental Results 7](#_Toc62332075)

[*Computational Modeling Results* 7](#_Toc62332076)

[*Potential Confounds* 8](#_Toc62332077)

[Supplemental Figures 12](#_Toc62332078)

[Supplemental Tables 20](#_Toc62332079)

## ***Pilot Study***

## ***Introduction***

The Novelty Task used in the current study is a variant of a task previously used only with adult participants (1–3). Given that our study was to be conducted with adolescents, we performed a pilot study with N=76 participants who were not included in the main sample. The purposes of this pilot study were to: (i) determine whether the Novelty task elicited PE signaling in an adolescent sample; (ii) determine whether our calculated novelty propensity was associated with enhanced RPE in “Explore” trials; and (iii) use the data obtained to make data-driven predictions for the main study. On the basis of previous work with adults, we predicted that: (i) regions of ventromedial prefrontal cortex (vmPFC), anterior cingulate cortex (ACC), dorsolateral prefrontal cortex (dlPFC), anterior insular cortex (aIC) posterior cingulate cortex (PCC), and striatum would be associated with RPE modulation relative to baseline; and (ii) these regions would show enhanced RPE modulation on Explore trials as a function of novelty propensity.

## ***Methods***

***Participants***

Participants for the pilot study included 76 healthy participants from the Omaha community. The exclusion criteria and consent procedures for the pilot study were identical to those described for the main study.

***Novelty Task***

Participants completed the Novelty task during fMRI scanning that was identical to the one described in the main study. The computational modeling procedures were identical to those used in the main study.

***MRI Parameters and fMRI Analysis***

The MRI procedures, fMRI preprocessing, and fMRI first-level GLM pipeline for the pilot study were identical to those described in the main study, except that clusters were considered significant at a more lenient threshold of initial *p*=.005 and extent threshold *k*=20 voxels. For the group level analysis, we conducted: (i) a one-sample *t*-test of RPE modulation versus baseline averaged across all trials; and (ii) a one-way ANCOVA with one within-subjects factor (Trial: Explore, Non-Explore) and novelty propensity as a covariate.

## ***Results***

***Demographic Data***

The average age of the sample used in the pilot study was 13.2 (SD=2.23). The average IQ was 109.6 (SD=13.67). There were 41 males and 35 females.

***fMRI Results***

**Main Effect of RPE Modulation:** RPE was directly associated with BOLD response modulation within regions including dlPFC, iFG, vmPFC, ACC, iPL, and striatum, such that positive RPE was associated with greater BOLD response modulation (Figure S2, Table S1). RPE was also inversely associated with BOLD modulation within regions of dorsal ACC, dorsomedial prefrontal cortex, and precuneus (Table S1). See Table S1 for full results.

**Novelty Propensity-by-Explore Interaction:** There was a significant Novelty Propensity-by-Explore interaction in regions including rmPFC, vmPFC, iPL, and ventral striatum (Figure S3, Table S2). In all cases, greater Novelty Propensity was associated with greater RPE modulation on Explore trials. See Table S2 for full results.

## ***Discussion***

These data indicate that vmPFC, ACC, dlPFC, aIC, PCC, and striatum are sensitive to RPE within an adolescent sample. This is congruent with the adult literature (O'Doherty *et al.*, 2007; O’Doherty *et al.*, 2017), which suggests that these brain regions are associated with neural representation of RPE. Additionally, these data indicate there is greater RPE modulation in rmPFC, vmPFC, iPL, and ventral striatum after the exploration of novel stimuli in individuals with greater novelty propensity, which is consistent with the adult data on a similar task (3).

# ***Main Study: Supplemental Methods***

## ***Recruitment***

Youths recruited from Boys Town had been referred for behavioral and mental health problems, including substance use disorders. These procedures are documented in a number of previous studies from our lab (6–12). Participants from the community were recruited through flyers or social media. Clinical characterization was done through psychiatric interviews by licensed and board-certified child and adolescent psychiatrists with the participants and their parents/caregivers, to adhere closely to common clinical practice.

The Boys Town National Research Hospital institutional review board approved this study. A doctoral level researcher or a member of the clinical research team obtained written informed consent and assent. In all cases, youth had the right to decline participation at any time before or during the study. Participants were compensated $30 for completing an initial screen, $50 for completing the scan, plus 10% of their total winnings from the task.

Exclusion criteria for the broader project included IQ<75 assessed with the Wechsler Abbreviated Scale of Intelligence (WASI two-subtest form; Wechsler, 2011), pregnancy, non-psychiatric medical conditions that require the use of medication that may have psychotropic effects (e.g., beta blockers or steroids), current psychosis, pervasive developmental disorders, Tourette’s disorder, neurological disorders, presence of metallic objects in the body (e.g., metal plates, pacemakers, etc.), and claustrophobia. Current psychiatric conditions (other than psychotic disorders or pervasive developmental disorders) were not exclusionary. Use of psychotropic medications for psychiatric indications (e.g., stimulants, selective serotonin reuptake inhibitors) were not exclusory. However, participants on stimulant medication were asked to withhold medication on the day of scanning.

***Variable Transformation***

Skewness and kurtosis values for the AUDIT were 2.71 and 9.17, respectively. Therefore, a Rankit Transformation was applied to AUDIT scores to reduce skewness and kurtosis. Post-transformation, skewness and kurtsosis for AUDIT were 0.67 and -0.44, respectively. The skewness and kurtosis values for the CUDIT were 0.67 and -0.73, so no transformation was applied to the CUDIT. Rankit-transformed AUDIT scores and raw CUDIT scores were then z-scored; z-scored, rankit-transformed AUDIT scores and z-scored CUDIT scores were used in all analyses.

## ***Computational Modeling: Reinforcement Learning Model***

The two free parameters of the model (learning rate, $\alpha$; and inverse temperature, β) were estimated through a nonlinear optimization process by maximizing the likelihood of the actual choices of participants. The probability, *d*, of a choice, *I*, given a value, *V*, was calculated using the softmax rule:

$$P_{i}{dP}_{i}\left( t \right)= \frac{e^{\beta v_{i}(t)}}{\sum_{k=1}^{3} e^{\beta v_{ki}(t)}}P_{i}\left( t \right)= \frac{e^{\beta v_{i}(t)}}{\sum_{k=1}^{3} e^{\beta v_{i}(t)}}$$

where k indexes the three available choices. The log-likelihood was then calculated as follows:

$$ll=-\sum_{t=1}^{T} log\sum_{k=1}^{3} c_{k}(t)d_{k}d(t)$$

Where *c_k_(t)*=1 when the participant chooses option *k* in trial *t* and *c_k_(t)*=0 for all unchosen options per trial. The model, therefore, maximizes the choice probability *d_k_(t)* of the actual choices made by participants. *T* represents the number of trials in each run for each participant (140). The log-likelihood was minimized using fminsearch in matlab. The initial values for the learning rate $\alpha$ were drawn from the standard uniform distribution on the open interval (0,1). The initial values for the inverse temperature β were drawn from a standard uniform distribution. The values of the two free parameters ($\alpha$ and β) were set at the value for which the iteration resulted in the minimum log-likelihood. Based on a larger population of 290 youths who performed the task used here (including current participants), a learning rate of α=0.692 was established. The average learning rate for the sample used in the study was α=0.653 (SD=0.3156).

## ***Computational Modeling: Novelty Propensity***

To determine *novelty propensity*, we examined the proportion of times that participants selected the novel stimulus on the *second* trial after the introduction of the novel stimulus. Notably, participants were most likely to pick the novel stimulus on the second trial after its introduction (43.2% as opposed to 33-36% on all other trials after introduction; *t*s=5.77-8.73, *p*s<.001; See Figure 2 of the Main Text). Participants were not more likely to pick the novel stimulus on the first trial after its introduction relative to the third or more trial after introduction (36.0% as opposed to 33-36%; *t*s=0.24-1.87, *p*s>.05). As such, participants were most likely to explore the novel stimulus on the second trial after its introduction and we termed these trials “Explore” trials.

To determine an individual’s novelty propensity, a logistic regression was calculated for each individual; specifically, the EV of the best non-novel option (non-novel stimulus with the highest EV; abbreviated as EV_best_) was used to predict the probability of the participant choosing the novel stimulus on the second trial after a novel stimulus is presented (assuming that higher values of EV_best_ were associated with lower probabilities of choosing the novel stimulus). The novelty propensity for each individual was defined as the EV_best_ for which the participant had a probability of 0.5 of choosing the novel stimulus. In order to ensure that plausible values were calculated, if this calculated value was outside the range of expected values of the best non-novel option for a participant, then it was set to either the minimum EV_best_ (if less than the minimum; N=2) or the maximum EV_best_ (if greater than the maximum; N=13). Based on a larger population of 290 youths who performed the task (including current participants), the average novelty propensity was 0.216. In other words, the average participant had an EV of 0.216 for the best alternative on trials where they had a 50% likelihood of selecting the novel stimulus. Therefore, novel stimuli were assigned an EV of 0.216 on the first time they were selected. The average novelty propensity for the final 128 participants used in the main study was 0.206 (SD=0.0804).

To evaluate overall model fit, we ran zero-order correlations between model-predicted proportions of best/worst non-novel stimuli chosen and actual proportions of best/worst non-novel stimuli chosen. We also ran zero-order correlations between AUDIT scores and CUDIT scores and learning rate to ensure that there was no relationship between these measures and learning rate. Finally, we ran linear regressions of actual decision proportions on model-predicted proportions, sex, AUDIT scores, CUDIT scores, AUDIT-by-model prediction interaction, and CUDIT-by-model prediction interaction to determine the consistency of model fit across the distribution of AUDIT and CUDIT scores.

***Functional MRI Parameters and Analysis***

Whole-brain functional MRI data were acquired via a 3T MAGNETOM Skyra magnetic resonance imaging scanner (Siemens Medical Solutions). The total amount of time for each run of the task varied slightly based on participant reaction time. A total of 242-275 functional images were taken for each run with a T2* weighted gradient echo planar imaging (EPI) sequence (repetition time=2500 ms; echo time=27 ms; 240 mm field of view; 94x94 matrix; 90^o^ flip angle). Whole-brain coverage was obtained with 43 axial slices (thickness, 2.5 mm; voxel size 2.6x2.6x2.5 mm^3^). A high-resolution T1 anatomical scan (MP-RAGE, repetition time=2200 ms; echo time=2.48 ms; 230 mm field of view; 8^o^ flip angle; 256x208 matrix; thickness, 1 mm; voxel size .9x.9x1 mm^3^) in register with the EPI data set was obtained covering the whole brain with 176 axial slices.

# ***Main Study: Supplemental Results***

## ***Computational Modeling Results***

We observed highly significant correlations between proportion of best/worst non-novel stimuli chosen and model-predicted probability of best/worst non-novel stimuli chosen, respectively [*r*s=0.87-0.91, *p*s<.001], indicating excellent model fit (See Supplemental Figure S1). There was no relationship between AUDIT scores and learning rate or CUDIT scores and learning rate [*r*s=0.00-0.04, *p*s>.05].To investigate whether our model fit changed as a function of AUDIT or CUDIT scores, we also ran linear regressions of proportions of best/worst non-novel stimuli chosen and model-predicted probabilities of best/worst non-novel stimuli chosen with sex, AUDIT, CUDIT, AUDIT-by-model prediction interaction, and CUDIT-by-model prediction interaction included as regressors. We did not find any AUDIT-by-model prediction or CUDIT-by-model prediction interaction effects on proportion of best/worst non-novel stimuli chosen, indicating that degree of model fit was consistent over the distribution of AUDIT and CUDIT scores.

## ***Potential Confounds***

Since our sample reflected clinical reality there were a number of potential confounds, including co-morbid psychiatric conditions, prescribed psychotropic medication usage, smoking status, suppression effects, and inclusion of all potentially relevant interaction effects. We chose to run separate models for each confounding variable because including all 13 variables would substantially reduce the statistical power of our analyses. In addition, this allowed us to explore any confound specific interactions that might need greater consideration in future work.
 To rule out the possibility that pathology related to psychiatric co-morbidities influenced our results, our main analysis was repeated with each of the four most common psychiatric diagnoses as a covariate (ADHD, CD, MDD, GAD). Each of these analyses revealed results similar to the main analysis; more specifically, (i) the main effects of AUDIT score on RPE modulation in ventral putamen (albeit at a lenient threshold of *k*>10 voxels in the ADHD analysis), aIC/iFG, dlPFC were significant after controlling for each of these diagnostic categories, (ii) the AUDIT-by-Explore interaction effect on RPE modulation in caudate, ventral putamen, aIC/iFG, and dlPFC were significant after controlling for each of these diagnostic categories, and (iii) the CUDIT-by-Novelty Propensity-by-Explore interaction effect on RPE modulation in dmPFC and iPL were significant after controlling for each of these diagnostic categories (Tables S3-S6).

We also covaried for prescribed antipsychotic, antidepressant, and stimulant use. These analyses again revealed results similar to the main analysis. More specifically, (i) the main effect of AUDIT on RPE modulation in ventral putamen, aIC, iFG, and dlPFC remained significant, (ii) the AUDIT-by-Explore interaction effect on RPE modulation in ventral putamen, aIC, iFG, and dlPFC remained significant, while the AUDIT-by-Explore interaction effect on RPE modulation in caudate remained significant, and (iii) the CUDIT-by-Novelty Propensity-by-Explore interaction effect on RPE modulation in dmPFC and iPL remained significant after controlling for prescribed use of these medications (Tables S7-S9).

We also conducted an analysis removing all participants who endorsed current regular smoking. These results were again similar to the results in the main analysis. The main effect of AUDIT on RPE modulation in aIC, iFG, and dlPFC remained significant. The AUDIT-by-Explore interaction effect on RPE modulation in ventral putamen, aIC, iFG, and dlPFC remained significant. The CUDIT-by-Novelty Propensity-by-Explore interaction effect on RPE modulation in dmPFC and iPL were significant after removing smokers from the sample (Table S10).

To rule out suppression effects, we also conducted analyses investigating the effects of AUDIT on RPE modulation without covarying for CUDIT and CUDIT on RPE modulation without covarying for AUDIT. The main effect of AUDIT remained significant in dlPFC, while the AUDIT-by-Explore interaction remained significant within dlPFC and at lenient thresholds in ventral putamen and iFG (Table S11). The CUDIT-by-Novelty Propensity interaction effect on RPE modulation remained significant in iPL and the CUDIT-by-Novelty Propensity-by-Explore interaction effect remained significant in iPL and at a lenient threshold, dmPFC (Table S12).

We also re-ran the analysis including all interactions between variables. In this analysis, the main effect of AUDIT remained significant at a lenient threshold (*k*>10 voxels) within insula and cerebellum. The AUDIT-by-Explore interaction effect remained significant within ventral putamen, aIC, iFG, and at a lenient threshold, caudate. The CUDIT-by-Novelty Propensity and the CUDIT-by-Novelty Propensity-by-Explore interactions remained significant within cerebellum (Table S13).

The vast majority of participants with significant substance use histories in this study were members of the residential program and were subject to random drug testing for at least four weeks prior to scanning. However, participants with significant substance use histories were recruited from the community and not subject to random drug testing. We repeated the same analysis with these participants excluded with from the sample; the main effect of AUDIT remained significant within ventral putamen, aIC, iFG, and dlPFC. The AUDIT-by-Explore interaction remained significant in ventral putamen, caudate, aIC, and iFG. The CUDIT-by-Novelty Propensity interaction remained significant in iPL and cerebellum and the CUDIT-by-Novelty-by-Explore remained significant in dmPFC, iPL, and superior temporal gyrus (Table S14).

Given the significant neurodevelopment that occurs between ages 14 and 18, we repeated the same analysis controlling for age; the main effect of AUDIT remained significant within ventral putamen, aIC, iFG, and dlPFC. The AUDIT-by-Explore interaction remained significant in ventral putamen, caudate, aIC, dlPFC, and iFG. The CUDIT-by-Novelty Propensity interaction remained significant in iPL and cerebellum and the CUDIT-by-Novelty-by-Explore remained significant in iPL, superior temporal gyrus, and dmPFC (Table S15).

***Categorical Analysis***

In order to better understand the current results of the study, we also ran a categorical analysis, with groups created by median splits of AUDIT and CUDIT scores. The median AUDIT score was 1 and the median CUDIT score was 5.5. This resulted in four groups: low AUDIT/low CUDIT (n=36), low AUDIT/high CUDIT (n=18), high AUDIT/low CUDIT (n=28), and high AUDIT/high CUDIT (n=46). Demographics for these groups can be found in Table S16.

We conducted a repeated-measures ANCOVA on the BOLD response data modulated by RPE. The between subjects categorical variables were AUDIT group (high/low), CUDIT group (high/low), and sex (male/female) was included as a variable of no interest. NP was included as a covariate. The within subjects variable was Decision (Explore/Non-explore).

Results from this analysis largely mirrored the results from the main analysis with regard to the high/low AUDIT groups. We found a main effect of AUDIT group within aIC, iFG, dlPFC, iPL, and Cerebellum. We found an AUDIT group-by-Condition interaction effect within ventral putamen, caudate, aIC, and dlPFC. We did not find any CUDIT group-by-NP interaction effects or CUDIT group-by-NP-by-Condition interactions. For further details see Table S16 and Figure S4.

# ***Supplemental Figures***

| 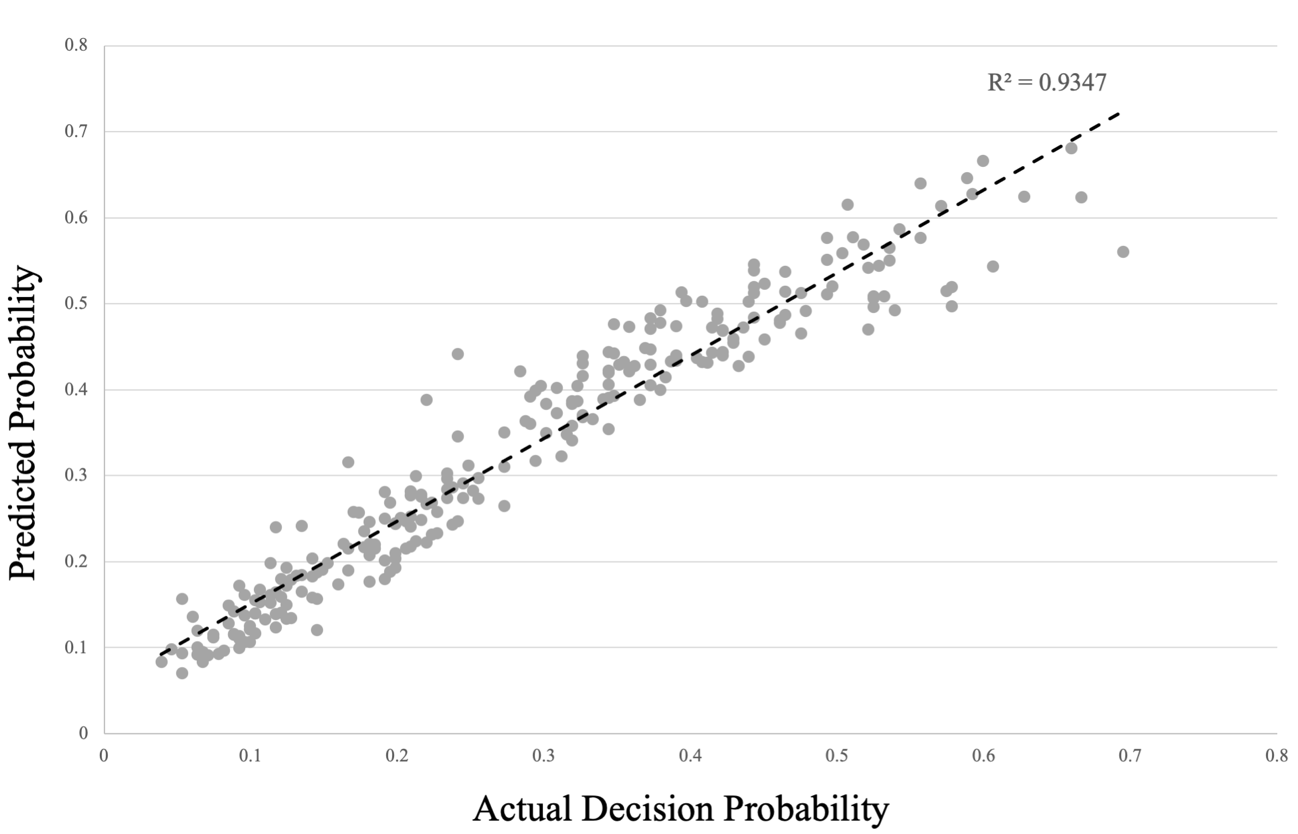 |
| --- |

Figure S1. Model-predicted proportions of best/worst non-novel stimuli chosen and actual proportions of best/worst non-novel stimuli chosen. The correlation between model-predicted decisions and actual decisions was *r*=0.967.

| 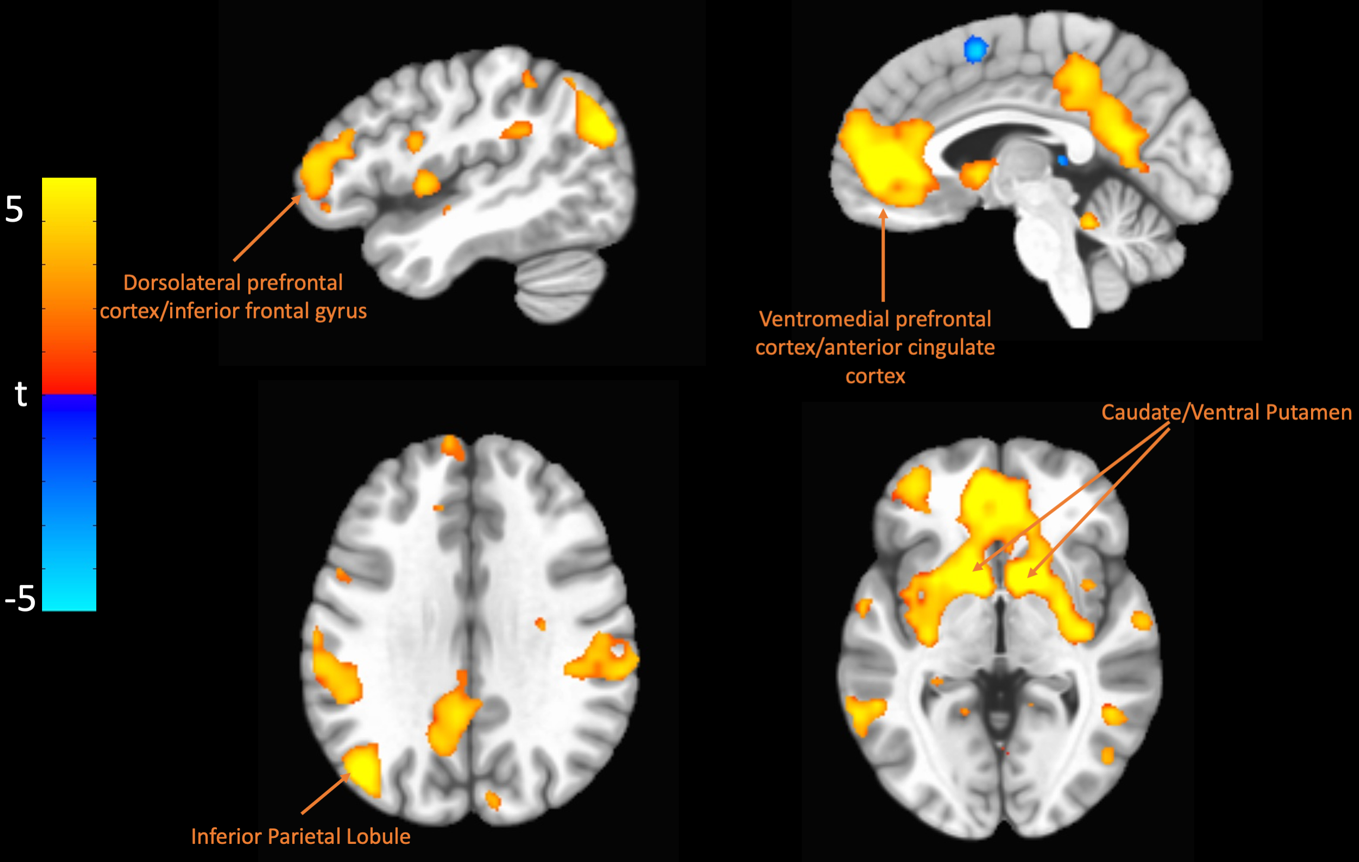 |
| --- |

Figure S2. Main effect of BOLD response modulated by RPE averaged across all trials in the Pilot Study. Notably, dorsolateral prefrontal cortex, inferior frontal gyrus, ventromedial prefrontal cortex, anterior cingulate cortex, inferior parietal lobule, and caudate/ventral putamen showed significant RPE modulation in the Pilot Study.

| 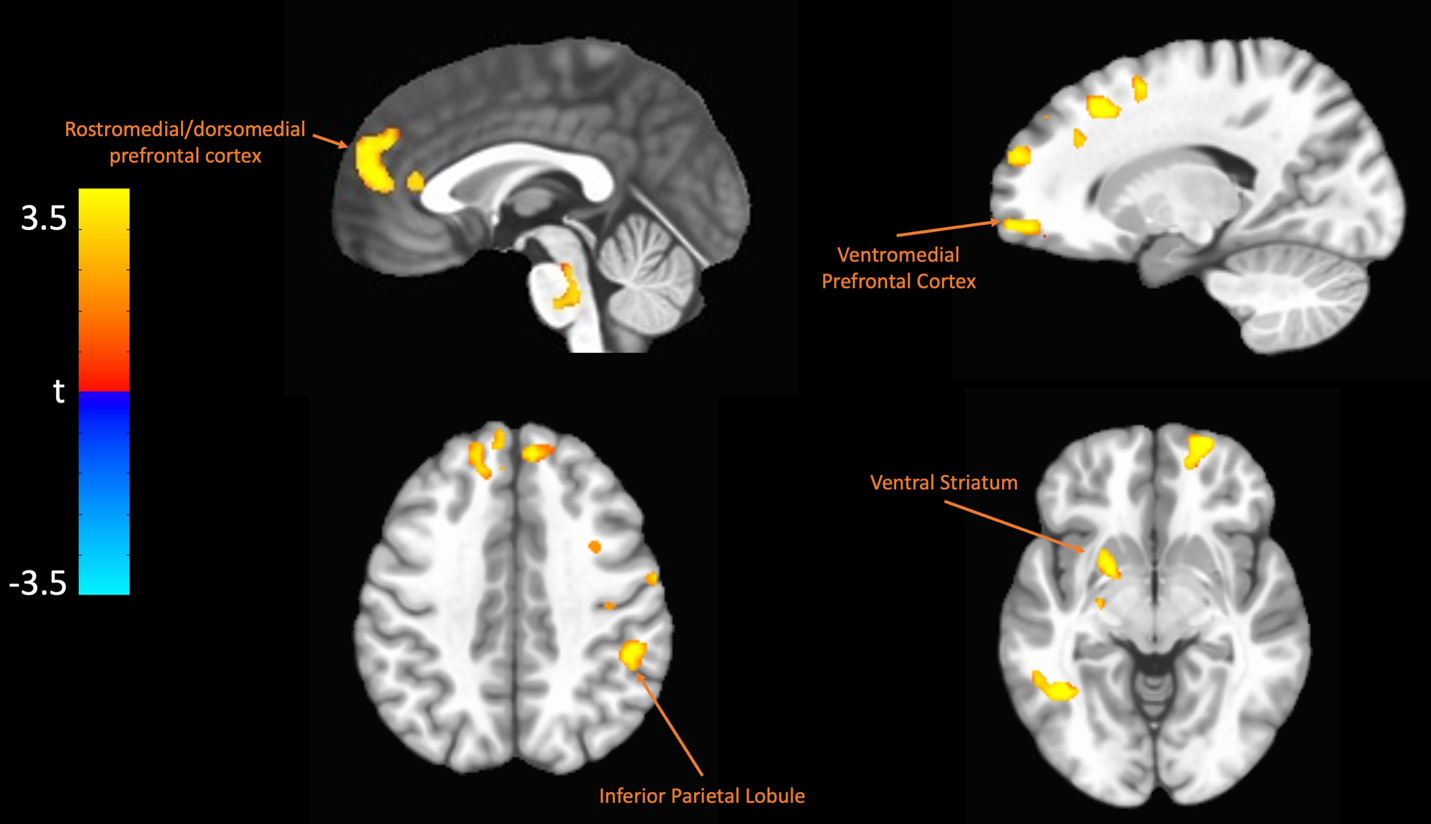 |
| --- |

Figure S3. Novelty Propensity-by-Explore interaction effect on BOLD response modulated by RPE. Notably, greater novelty propensity was associated with greater RPE modulation when exploring novel stimuli within rostromedial prefrontal cortex, dorsomedial prefrontal cortex, ventromedial prefrontal cortex, inferior parietal lobule, and ventral striatum

| 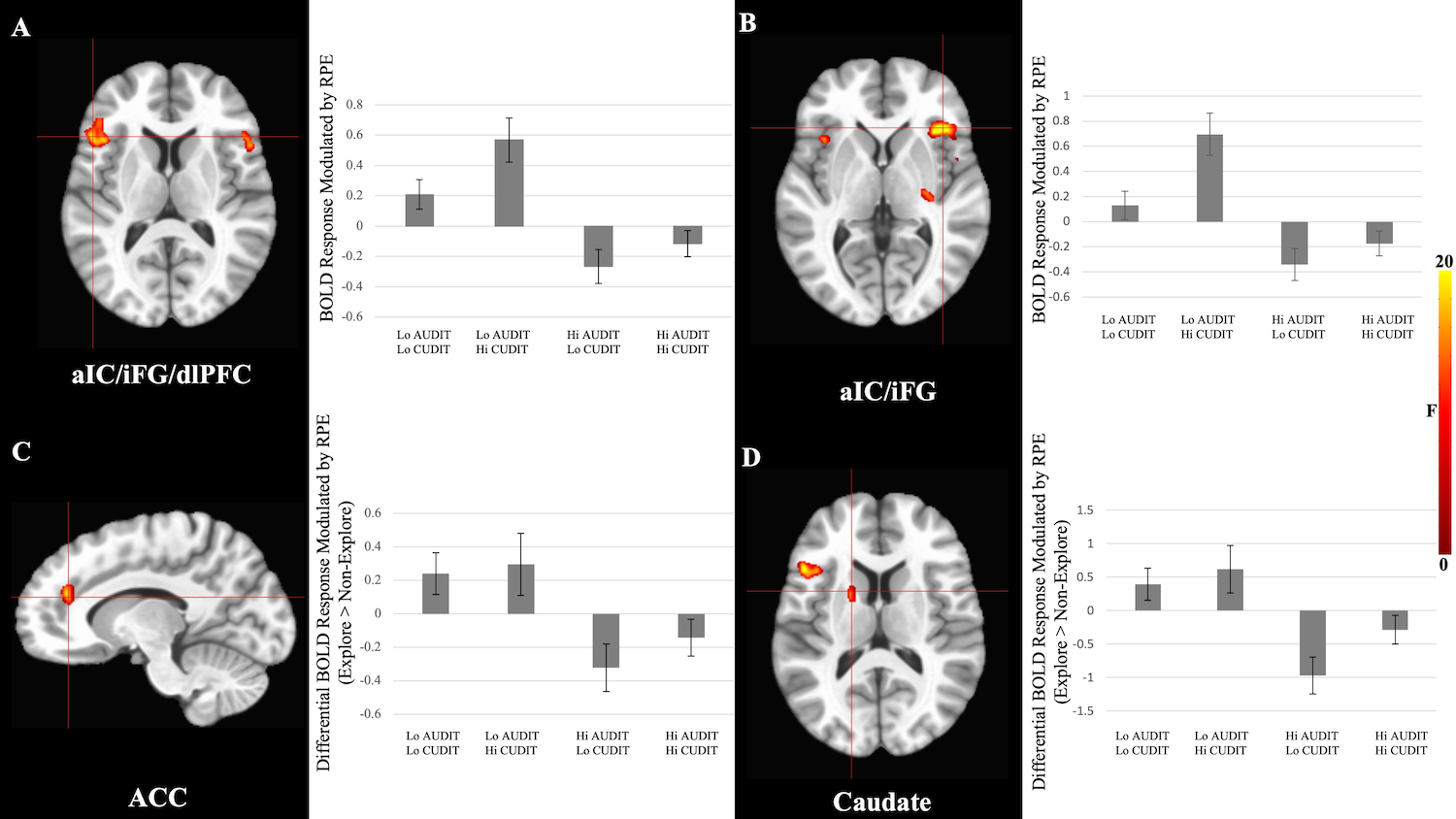 |
| --- |

Figure S4. AUDIT Group Main Effect in A) aIC/iFG/dlPFC and B) aIC/IFG; AUDIT Group by Explore Interaction Effect in C) ACC and D) Caudate. All clusters displayed overlap with clusters reported in the main analysis.

**Supplemental References**

1. Djamshidian A, O’Sullivan SS, Wittmann BC, Lees AJ, Averbeck BB (2011): Novelty seeking behaviour in Parkinson’s disease. *Neuropsychologia*. 49: 2483–2488.

2. Averbeck BB, Djamshidian A, O’Sullivan SS, Housden CR, Roiser JP, Lees AJ (2013): Uncertainty about mapping future actions into rewards may underlie performance on multiple measures of impulsivity in behavioral addiction: Evidence from Parkinson’s disease. *Behav Neurosci*. 127: 245–255.

3. Wittmann BC, Daw ND, Seymour B, Dolan RJ (2008): Striatal Activity Underlies Novelty-Based Choice in Humans. *Neuron*. 58: 967–973.

4. O’Doherty JP, Cockburn J, Pauli WM (2017): Learning, Reward, and Decision Making. *Annu Rev Psychol*. 68: 73–100.

5. O’DOHERTY JP, HAMPTON A, KIM H (2007): Model-Based fMRI and Its Application to Reward Learning and Decision Making. *Ann N Y Acad Sci*. 1104: 35–53.

6. Blair KS, Aloi J, Crum K, Meffert H, White SF, Taylor BK, *et al.* (2019): Association of Different Types of Childhood Maltreatment With Emotional Responding and Response Control Among Youths. *JAMA Netw Open*. 2: 1–15.

7. Crum KI, Hwang S, Blair RJR, Blair KS, Aloi JM, Meffert H, *et al.* (2020): Interaction of irritability and anxiety on emotional responding and emotion regulation : a functional MRI study. *Psychol Med*. 1–11.

8. Aloi J, Meffert H, White SF, Blair KS, Hwang S, Tyler PM, *et al.* (2019): Differential dysfunctions related to alcohol and cannabis use disorder symptoms in reward and error-processing neuro-circuitries in adolescents. *Dev Cogn Neurosci*. 36. doi: 10.1016/j.dcn.2019.100618.

9. Leiker EK, Meffert H, Thornton LC, Taylor BK, Aloi J, Abdel-Rahim H, *et al.* (2019): Alcohol Use Disorder and Cannabis Use Disorder symptomatology in adolescents are differentially related to dysfunction in brain regions supporting face processing. *Psychiatry Res Neuroimaging*. 292. doi: 10.1016/j.pscychresns.2019.09.004.

10. Aloi J, Blair KS, Crum KI, Bashford-Largo J, Zhang R, Lukoff J, *et al.* (2020): Alcohol Use Disorder, But Not Cannabis Use Disorder, Symptomatology in Adolescents Is Associated With Reduced Differential Responsiveness to Reward Versus Punishment Feedback During Instrumental Learning. *Biol Psychiatry Cogn Neurosci Neuroimaging*. 5: 610–618.

11. Aloi J, Blair KS, Crum KI, Meffert H, White SF, Tyler PM, *et al.* (2018): Adolescents show differential dysfunctions related to Alcohol and Cannabis Use Disorder severity in emotion and executive attention neuro-circuitries. *NeuroImage Clin*. 19: 782–792.

12. Aloi J, Blair KS, Meffert H, White SF, Hwang S, Tyler PM, *et al.* (2020): Alcohol Use Disorder and Cannabis Use Disorder symptomatology in adolescents is associated with dysfunction in neural processing of future events. *Addict Biol*.

13. Wechsler D (2011): *Wechsler Abbreviated Scale of Intelligence–Second Edition*. San Antonio, TX: NCS Pearson.

14. Cox RW (1996): AFNI: Software for Analysis and Visualization of Functional Magnetic Resonance Neuroimages. *Comput Biomed Res*. 29: 162–173.

15. Talairach J, Tournoux P (1988): *Co-Planar Stereotaxic Atlas of the Human Brain: 3-D Proportional System: An Approach to Cerebral Imaging*, 1st ed. Stuttgart: Thieme.

16. Cox RW, Chen G, Glen DR, Reynolds RC, Taylor PA (2017): FMRI Clustering in AFNI: False-Positive Rates Redux. *Brain Connect*. 7: 152–171.

# ***Supplemental Tables***

| Table S1. Brain regions demonstrating main effects of RPE modulation averaged across all trials in the pilot group | | | | | | | | |
| --- | --- | --- | --- | --- | --- | --- | --- | --- |
| Coordinates of Peak Activation^b^ | | | | | | | | |
| Region^a^ | Hemisphere | BA | x | y | z | *F* | Partial η^2^ | Voxels |
| PE > Baseline | | | | | | | | |
| Striatum/amygdala/vmPFC/ACC/dlPFC/iPL | R/L | 24/32/46/10/13/47/40 | 14 | 11 | -1 | 65.79 | 0.467 | 2780 |
| iFG/dlPFC | L | 11/47 | -34 | 41 | -4 | 33.04 | 0.306 | 227 |
| Middle/Superior Frontal Gyrus | L | 6 | -22 | 14 | 56 | 23.10 | 0.235 | 122 |
| ACC/Mid-Cingulate Cortex | R | 24 | 8 | -4 | 44 | 20.55 | 0.215 | 28 |
| Posterior Cingulate Cortex/Precuneus | R/L | 30 | -7 | -52 | 14 | 37.94 | 0.336 | 903 |
| Postcentral Gyrus | L | 5 | -22 | -40 | 65 | 15.69 | 0.173 | 21 |
| Superior Temporal Gyrus | R | 22/42 | 59 | -31 | 14 | 36.41 | 0.327 | 339 |
| Superior Temporal Gyrus | R | 21/22 | 59 | -7 | 2 | 26.00 | 0.257 | 33 |
| Middle Temporal Gyrus | L | 37/21 | -55 | -46 | -4 | 30.63 | 0.290 | 89 |
| Middle Temporal Gyrus | R | 21/22 | 47 | -46 | 2 | 26.96 | 0.264 | 74 |
| Cuneus | R | 18/19 | 8 | -82 | 23 | 15.76 | 0.174 | 34 |
| Cerebellum | R/L | - | -4 | -40 | -19 | 25.86 | 0.256 | 92 |
| Cerebellum | R | - | 26 | -58 | -34 | 18.93 | 0.202 | 44 |
| Parahippocampal Gyrus | R | 36 | 29 | -31 | -10 | 28.56 | 0.276 | 27 |
| Baseline > PE | | | | | | | | |
| dmPFC | R/L | 6 | -4 | 5 | 50 | 19.40 | 0.205 | 102 |
| Middle Frontal Gyrus | R | 6 | 35 | -1 | 47 | 15.82 | 0.174 | 22 |
| Precuneus | R | 7 | 8 | -61 | 44 | 19.98 | 0.210 | 57 |
| Parahippocampal Gyrus | R/L | - | 5 | -34 | 2 | 15.19 | 0.168 | 22 |

Note: ^a^ According to the Talairach Daemon Atlas (<http://www.nitrc.org/projects/tal-daemon/>), ^b^ Based on
the Tournoux & Talairach standard brain template, BA= Brodmann’s Area

| Table S2. Brain regions demonstrating Novelty Propensity-by-Explore interaction effects in the pilot group | | | | | | | | |
| --- | --- | --- | --- | --- | --- | --- | --- | --- |
| Coordinates of Peak Activation^b^ | | | | | | | | |
| Region^a^ | Hemisphere | BA | x | y | z | *F* | Partial η^2^ | Voxels |
| Novelty Propensity-by-Explore Interaction | | | | | | | | |
| Ventral Putamen | L | - | -16 | 2 | -4 | 16.01 | 0.178 | 36 |
| Amygdala/Putamen | L | - | -28 | -10 | -10 | 17.36 | 0.190 | 33 |
| Amygdala/Superior Temporal Gyrus | L | 38 | -22 | 14 | -34 | 20.71 | 0.219 | 67 |
| Amygdala/Parahippocampal Gyrus | R | 34 | 32 | -1 | -19 | 18.19 | 0.197 | 20 |
| Brain Stem | R/L | - | -1 | -25 | -19 | 13.73 | 0.157 | 30 |
| rmPFC/dmPFC/ACC | R/L | 24 | 5 | 32 | 14 | 17.49 | 0.191 | 299 |
| vmPFC | R | 10 | 23 | 56 | -4 | 17.87 | 0.194 | 29 |
| dmPFC | R | 6 | 17 | 8 | 50 | 16.32 | 0.181 | 25 |
| dlPFC | L | 9/46 | -37 | 20 | 26 | 13.92 | 0.158 | 27 |
| dlPFC | R | 8 | 17 | 23 | 41 | 18.45 | 0.200 | 27 |
| dlPFC | R | 6/9 | 32 | 5 | 38 | 16.89 | 0.186 | 26 |
| dlPFC | R | 9 | 32 | 35 | 32 | 15.94 | 0.177 | 59 |
| Middle Frontal Gyrus | R | 46 | 50 | 23 | 23 | 16.81 | 0.185 | 36 |
| Middle Frontal Gyrus | L | 6 | -19 | 5 | 59 | 14.53 | 0.164 | 34 |
| Superior Frontal Gyrus/mPFC | R | 9/10 | 14 | 56 | 23 | 12.95 | 0.149 | 29 |
| Precentral Gyrus | L | 4 | -13 | -34 | 68 | 16.06 | 0.178 | 53 |
| Precentral Gyrus | R | 4 | 56 | -10 | 32 | 14.75 | 0.166 | 25 |
| Precentral Gyrus | R | 3/4 | 38 | -19 | 35 | 16.40 | 0.181 | 24 |
| Mid-Cingulate Cortex | R | 24 | 11 | -19 | 44 | 12.87 | 0.148 | 22 |
| iFG | R | 47 | 38 | 20 | -19 | 16.96 | 0.186 | 21 |
| Parahippocampal Gyrus | L | 19 | -37 | -49 | -4 | 19.89 | 0.212 | 48 |
| Supramarginal Gyrus/iPL | R | 40 | 47 | -37 | 35 | 14.40 | 0.163 | 41 |
| Middle Temporal Gyrus | L | 19 | -37 | -61 | 11 | 16.13 | 0.179 | 42 |

Note: ^a^ According to the Talairach Daemon Atlas (<http://www.nitrc.org/projects/tal-daemon/>), ^b^ Based on
the Tournoux & Talairach standard brain template, BA= Brodmann’s Area

| Table S3. Brain regions demonstrating significant AUDIT, AUDIT-by-Explore, CUDIT-by-Novelty Propensity, and CUDIT-by-Novelty Propensity-by-Explore effects controlling for ADHD Diagnosis | | | | | | | | |
| --- | --- | --- | --- | --- | --- | --- | --- | --- |
| Coordinates of Peak Activation^b^ | | | | | | | | |
| Region^a^ | Hemisphere | BA | x | y | z | *F* | Partial η^2^ | Voxels |
| Main Effect of AUDIT | | | | | | | | |
| Ventral Putamen*^c^ | R | - | 26 | 14 | -1 | 17.44 | 0.127 | 11 |
| Putamen^c^ | R | - | 29 | -19 | 2 | 19.19 | 0.137 | 15 |
| dlPFC* | R | 10 | 29 | 50 | 26 | 17.72 | 0.129 | 26 |
| dlPFC* | L | 10/46 | -37 | 44 | 5 | 17.51 | 0.127 | 25 |
| dlPFC | L | 10 | -22 | 44 | 26 | 16.28 | 0.119 | 17 |
| aIC/iFG* | R | 22/45/ 47/13 | 53 | -1 | 2 | 30.93 | 0.205 | 120 |
| aIC/iFG* | L | 45 | -46 | 20 | 11 | 22.80 | 0.160 | 49 |
| iFG* | R | 11 | 38 | 35 | -10 | 25.21 | 0.174 | 21 |
| Precentral Gyrus^c^ | L | 6 | -55 | 2 | 29 | 20.69 | 0.146 | 13 |
| Cingulate Gyrus^c^ | L | 24 | -1 | -13 | 35 | 17.34 | 0.125 | 11 |
| iPL* | L | 40 | -49 | -49 | 32 | 18.52 | 0.134 | 24 |
| Cerebellum* | R | - | 32 | -52 | -37 | 25.84 | 0.177 | 22 |
| Cerebellum* | L | - | -31 | -49 | -28 | 27.05 | 0.184 | 40 |
| Thalamus^c^ | R | - | 8 | -25 | 8 | 14.62 | 0.108 | 11 |
| AUDIT-by-Explore | | | | | | | | |
| Caudate* | L | - | -16 | 5 | 11 | 15.98 | 0.118 | 21 |
| Ventral Putamen/aIC/iFG * | R | 47 | 47 | 17 | 2 | 24.80 | 0.171 | 78 |
| aIC/iFG* | L | 45 | -46 | 20 | 11 | 18.02 | 0.131 | 23 |
| dlPFC* | L | 10/46 | -37 | 44 | 5 | 18.51 | 0.134 | 27 |
| ACC*^c^ | L | 32 | -10 | 35 | 17 | 16.21 | 0.119 | 14 |
| iFG* | R | 11 | 38 | 35 | -7 | 21.62 | 0.153 | 21 |
| Supramarginal Gyrus | L | 40 | -49 | -52 | 35 | 16.48 | 0.121 | 21 |
| CUDIT-by-Novelty Propensity | | | | | | | | |
| iPL* | R | 40 | 47 | -40 | 38 | 20.92 | 0.148 | 42 |
| Cerebellum* | R/L | - | 8 | -79 | -25 | 17.83 | 0.129 | 22 |
| CUDIT-by-Novelty Propensity-by-Explore | | | | | | | | |
| dmPFC* | R/L | 32 | 2 | 20 | 41 | 17.34 | 0.126 | 21 |
| iPL* | R | 40 | 47 | -40 | 38 | 24.17 | 0.168 | 31 |
| Superior Temporal Gyrus* | R | 13 | 59 | -43 | 17 | 18.66 | 0.135 | 26 |

Note: ^a^ According to the Talairach Daemon Atlas (<http://www.nitrc.org/projects/tal-daemon/>), ^b^ Based on
the Tournoux & Talairach standard brain template, ^c^ Below the ClustSim established threshold, BA= Brodmann’s Area,
* Overlapping or proximal to a significant cluster in the main analysis

| Table S4. Brain regions demonstrating significant AUDIT, AUDIT-by-Explore, CUDIT-by-Novelty Propensity, and CUDIT-by-Novelty Propensity-by-Explore effects controlling for CD Diagnosis | | | | | | | | |
| --- | --- | --- | --- | --- | --- | --- | --- | --- |
| Coordinates of Peak Activation^b^ | | | | | | | | |
| Region^a^ | Hemisphere | BA | x | y | z | *F* | Partial η^2^ | Voxels |
| Main Effect of AUDIT | | | | | | | | |
| Ventral Putamen/aIC/iFG* | R | 22/45/ 47/13 | 47 | 14 | 5 | 33.10 | 0.216 | 199 |
| Putamen | R | - | 29 | -22 | 2 | 20.11 | 0.144 | 18 |
| dlPFC* | R | 9/10 | 29 | 50 | 23 | 20.57 | 0.146 | 50 |
| dlPFC* | L | 10/46 | -37 | 44 | 5 | 19.03 | 0.137 | 39 |
| dlPFC | L | 10 | -22 | 44 | 26 | 17.46 | 0.127 | 19 |
| aIC/iFG* | L | 45 | -46 | 20 | 11 | 22.58 | 0.158 | 52 |
| iFG* | R | 11 | 38 | 35 | -10 | 24.22 | 0.168 | 22 |
| Precentral Gyrus^c^ | L | 6 | -55 | 2 | 29 | 20.32 | 0.144 | 13 |
| MCC | R/L | 23 | -1 | -13 | 32 | 21.00 | 0.149 | 21 |
| Supramarginal Gyrus* | L | 40 | -49 | -52 | 35 | 20.26 | 0.144 | 29 |
| Cerebellum* | L | - | -31 | -49 | -28 | 25.45 | 0.175 | 36 |
| Cerebellum* | R | - | 32 | -52 | -37 | 24.72 | 0.171 | 21 |
| Cerebellum^c^ | L | - | -4 | -64 | -13 | 13.73 | 0.102 | 11 |
| Thalamus | R | - | 8 | -22 | 5 | 27.23 | 0.185 | 25 |
| AUDIT-by-Explore | | | | | | | | |
| Caudate* | L | - | -16 | 5 | 11 | 17.24 | 0.126 | 24 |
| Ventral Putamen/aIC/iFG* | R | 47 | 26 | 14 | -1 | 28.59 | 0.192 | 129 |
| aIC/iFG* | L | 13/45 | -43 | 20 | 11 | 17.78 | 0.129 | 19 |
| aIC/iFG*^c^ | L | 45 | -25 | 26 | 2 | 15.96 | 0.117 | 10 |
| aIC/iFG*^c^ | L | 13/47 | -34 | 17 | 2 | 14.71 | 0.108 | 10 |
| dlPFC* | L | 10/46 | -37 | 44 | 5 | 21.92 | 0.154 | 61 |
| ACC* | L | 32 | -10 | 35 | 17 | 16.91 | 0.123 | 22 |
| iFG* | R | 11 | 38 | 35 | -7 | 19.97 | 0.143 | 19 |
| Supramarginal Gyrus* | L | 40 | -49 | -52 | 35 | 16.99 | 0.124 | 24 |
| Thalamus | R | - | 17 | -22 | -1 | 21.45 | 0.151 | 14 |
| Claustrum | L | 13 | -34 | 2 | 2 | 13.22 | 0.099 | 10 |
| CUDIT-by-Novelty Propensity | | | | | | | | |
| iPL* | R | 40 | 47 | -40 | 38 | 18.09 | 0.131 | 53 |
| Cerebellum* | R/L | - | 5 | -79 | -25 | 19.99 | 0.143 | 30 |
| CUDIT-by-Novelty Propensity-by-Explore | | | | | | | | |
| dmPFC* | R/L | 32 | 2 | 20 | 41 | 17.44 | 0.127 | 20 |
| MCC | R/L | 24 | 2 | -13 | 38 | 21.94 | 0.155 | 19 |
| iPL* | R | 40 | 47 | -40 | 38 | 19.51 | 0.140 | 35 |
| Superior Temporal Gyrus | R | 13 | 59 | -43 | 17 | 24.47 | 0.169 | 27 |

Note: ^a^ According to the Talairach Daemon Atlas (<http://www.nitrc.org/projects/tal-daemon/>), ^b^ Based on
the Tournoux & Talairach standard brain template, ^c^ Below the ClustSim established threshold, BA= Brodmann’s Area,
*Overlapping with or proximal to a cluster reported in the main analysis

| Table S5. Brain regions demonstrating significant AUDIT, AUDIT-by-Explore, CUDIT-by-Novelty Propensity, and CUDIT-by-Novelty Propensity-by-Explore effects controlling for MDD Diagnosis | | | | | | | | |
| --- | --- | --- | --- | --- | --- | --- | --- | --- |
| Coordinates of Peak Activation^b^ | | | | | | | | |
| Region^a^ | Hemisphere | BA | x | y | z | *F* | Partial η^2^ | Voxels |
| Main Effect of AUDIT | | | | | | | | |
| Ventral Putamen/aIC/iFG* | R | 22/45/ 47/13 | 53 | -1 | 2 | 31.57 | 0.208 | 143 |
| Putamen^c^ | R | - | 29 | -19 | 2 | 19.27 | 0.137 | 15 |
| dlPFC* | L | 10/46 | -37 | 44 | 5 | 20.84 | 0.148 | 57 |
| dlPFC* | R | 9/10 | 29 | 50 | 26 | 25.51 | 0.175 | 27 |
| dlPFC*^c^ | L | 10 | -22 | 44 | 26 | 18.03 | 0.130 | 16 |
| aIC/iFG* | L | 45 | -46 | 20 | 11 | 26.41 | 0.180 | 67 |
| iFG* | R | 11 | 38 | 35 | -10 | 25.62 | 0.176 | 24 |
| Precentral Gyrus^c^ | L | 6 | -55 | 2 | 29 | 18.90 | 0.135 | 13 |
| Supramarginal Gyrus* | L | 40 | -49 | -49 | 35 | 17.34 | 0.126 | 37 |
| Fusiform Gyrus^c^ | R | 37 | 50 | -43 | -16 | 18.07 | 0.130 | 14 |
| Superior Temporal Gyrus | R | 22 | 53 | -1 | 2 | 19.79 | 0.142 | 41 |
| Cerebellum* | L | - | -31 | -49 | -28 | 27.14 | 0.184 | 42 |
| Cerebellum* | R | - | 32 | -52 | -37 | 16.53 | 0.121 | 20 |
| AUDIT-by-Explore | | | | | | | | |
| Caudate* | L | - | -16 | 5 | 11 | 15.67 | 0.115 | 19 |
| Ventral Putamen/aIC/iFG* | R | 45 | 35 | 26 | 5 | 25.06 | 0.173 | 88 |
| aIC/iFG* | L | 45 | -46 | 20 | 11 | 21.97 | 0.155 | 48 |
| dlPFC* | L | 10/46 | -37 | 44 | 5 | 23.31 | 0.163 | 69 |
| iFG* | R | 11 | 38 | 35 | -7 | 23.46 | 0.164 | 24 |
| ACC*^c^ | L | 32 | -10 | 35 | 17 | 16.03 | 0.118 | 15 |
| dmPFC^c^ | L | 8 | -4 | 23 | 47 | 16.17 | 0.118 | 12 |
| iPL | L | 40 | -49 | -55 | 38 | 16.12 | 0.118 | 20 |
| CUDIT-by-Novelty Propensity | | | | | | | | |
| iPL* | R | 40 | 47 | -40 | 38 | 20.87 | 0.148 | 38 |
| Cerebellum* | R/L | - | 8 | -79 | -25 | 19.67 | 0.141 | 19 |
| CUDIT-by-Novelty Propensity-by-Explore | | | | | | | | |
| dmPFC* | R/L | 32 | 2 | 20 | 41 | 17.55 | 0.128 | 21 |
| iPL* | R | 40 | 47 | -40 | 38 | 18.34 | 0.133 | 30 |
| Superior Temporal Gyrus | R | 13 | 59 | -43 | 17 | 23.81 | 0.166 | 25 |

Note: ^a^ According to the Talairach Daemon Atlas (<http://www.nitrc.org/projects/tal-daemon/>), ^b^ Based on
the Tournoux & Talairach standard brain template, ^c^ Below the ClustSim established threshold, BA= Brodmann’s Area,
* Overlapping with or proximal to a cluster reported in the main analysis

| Table S6. Brain regions demonstrating significant AUDIT, AUDIT-by-Explore, CUDIT-by-Novelty Propensity, and CUDIT-by-Novelty Propensity-by-Explore effects controlling for GAD Diagnosis | | | | | | | | |
| --- | --- | --- | --- | --- | --- | --- | --- | --- |
| Coordinates of Peak Activation^b^ | | | | | | | | |
| Region^a^ | Hemisphere | BA | x | y | z | *F* | Partial η^2^ | Voxels |
| Main Effect of AUDIT | | | | | | | | |
| Ventral Putamen/aIC/iFG* | R | 45/47/13 | 53 | -1 | 2 | 31.78 | 0.209 | 129 |
| Putamen | R | - | 29 | -19 | 2 | 19.53 | 0.139 | 15 |
| dlPFC* | L | 10/46 | -37 | 44 | 5 | 18.02 | 0.131 | 30 |
| dlPFC* | R | 9/10 | 29 | 50 | 26 | 17.45 | 0.127 | 26 |
| dlPFC | L | 9 | -22 | 44 | 26 | 16.70 | 0.122 | 18 |
| aIC/iFG* | L | 32 | -46 | 20 | 11 | 23.11 | 0.161 | 51 |
| iFG* | R | 11 | 38 | 35 | -10 | 24.63 | 0.170 | 20 |
| Precentral Gyrus | L | 6 | -55 | 2 | 29 | 19.77 | 0.140 | 13 |
| Supramarginal Gyrus* | L | 40 | -49 | -49 | 32 | 17.34 | 0.126 | 24 |
| Cerebellum* | L | - | -31 | -49 | -28 | 27.09 | 0.184 | 39 |
| Cerebellum* | R | - | 32 | -52 | -37 | 25.51 | 0.175 | 21 |
| Thalamus | R | - | 8 | -25 | 8 | 19.65 | 0.140 | 10 |
| AUDIT-by-Explore | | | | | | | | |
| Caudate* | L | - | -16 | 5 | 11 | 16.13 | 0.118 | 22 |
| Ventral Putamen/aIC/iFG* | R | 47 | 47 | 17 | 2 | 25.16 | 0.173 | 81 |
| aIC/iFG* | L | 45 | -46 | 20 | 11 | 18.56 | 0.134 | 24 |
| dlPFC* | L | 10/46 | -37 | 44 | 5 | 19.11 | 0.137 | 36 |
| ACC*^c^ | L | 32 | -7 | 32 | 26 | 15.93 | 0.117 | 14 |
| iFG* | R | 47 | 35 | 32 | -4 | 20.87 | 0.148 | 22 |
| iPL | L | 40 | -49 | -55 | 38 | 16.02 | 0.118 | 18 |
| CUDIT-by-Novelty Propensity | | | | | | | | |
| iPL* | R | 40 | 47 | -40 | 38 | 21.24 | 0.150 | 39 |
| Cerebellum* | R/L | - | 5 | -76 | -28 | 19.97 | 0.143 | 20 |
| CUDIT-by-Novelty Propensity-by-Explore | | | | | | | | |
| dmPFC* | R/L | 32 | 2 | 20 | 41 | 20.22 | 0.144 | 21 |
| iPL* | R | 40 | 47 | -40 | 38 | 23.93 | 0.166 | 32 |
| Superior Temporal Gyrus | R | 13 | 59 | -43 | 17 | 17.68 | 0.128 | 25 |

Note: ^a^ According to the Talairach Daemon Atlas (<http://www.nitrc.org/projects/tal-daemon/>), ^b^ Based on
the Tournoux & Talairach standard brain template, ^c^ Below the ClustSim established threshold, BA= Brodmann’s Area,
*Overlapping with or proximal to a cluster reported in the main analysis

| Table S7. Brain regions demonstrating significant AUDIT, AUDIT-by-Explore, CUDIT-by-Novelty Propensity, and CUDIT-by-Novelty Propensity-by-Explore effects covarying for antipsychotic use | | | | | | | | |
| --- | --- | --- | --- | --- | --- | --- | --- | --- |
| Coordinates of Peak Activation^b^ | | | | | | | | |
| Region^a^ | Hemisphere | BA | x | y | z | *F* | Partial η^2^ | Voxels |
| Main Effect of AUDIT | | | | | | | | |
| Ventral Putamen/aIC/iFG* | R | 45/47/13 | 53 | -1 | 2 | 29.71 | 0.198 | 81 |
| Putamen^c^ | R | - | 29 | -19 | 2 | 18.54 | 0.133 | 14 |
| dlPFC* | R | 9/10 | 29 | 50 | 23 | 18.09 | 0.131 | 26 |
| dlPFC* | L | 10/46 | -37 | 44 | 5 | 16.92 | 0.124 | 21 |
| dlPFC^c^ | L | 10 | -22 | 44 | 26 | 17.38 | 0.126 | 14 |
| aIC/iFG* | L | 32 | -46 | 20 | 11 | 22.14 | 0.156 | 37 |
| iFG* | R | 11 | 38 | 35 | -10 | 21.10 | 0.148 | 17 |
| Cingulate Gyrus^c^ | R | 24 | -1 | -13 | 35 | 18.31 | 0.131 | 12 |
| Precentral Gyrus^c^ | L | 6/9 | -55 | 2 | 29 | 18.60 | 0.133 | 11 |
| Supramarginal Gyrus* | L | 40 | -49 | -49 | 32 | 17.28 | 0.126 | 21 |
| Cerebellum* | L | - | -31 | -49 | -28 | 26.80 | 0.183 | 40 |
| AUDIT-by-Explore | | | | | | | | |
| Caudate*^c^ | L | - | -16 | 5 | 11 | 15.25 | 0.113 | 11 |
| Ventral Putamen/aIC/iFG* | R | 47 | 47 | 17 | 2 | 24.08 | 0.167 | 66 |
| aIC/iFG*^c^ | L | 45 | -46 | 20 | 11 | 17.23 | 0.126 | 14 |
| dlPFC* | L | 10/46 | -37 | 44 | 5 | 17.69 | 0.128 | 21 |
| ACC*^c^ | L | 32 | -10 | 35 | 17 | 20.19 | 0.144 | 14 |
| iFG*^c^ | R | 47 | 38 | 35 | -7 | 15.76 | 0.116 | 13 |
| iPL | L | 40 | -49 | -55 | 38 | 15.84 | 0.117 | 16 |
| CUDIT-by-Novelty Propensity | | | | | | | | |
| iPL* | R | 40 | 47 | -40 | 38 | 21.00 | 0.149 | 39 |
| Cerebellum* | R/L | - | 5 | -76 | -28 | 19.64 | 0.141 | 19 |
| CUDIT-by-Novelty Propensity-by-Explore | | | | | | | | |
| dmPFC* | R/L | 32 | 2 | 20 | 41 | 17.77 | 0.129 | 22 |
| iPL* | R | 40 | 47 | -40 | 38 | 18.39 | 0.133 | 31 |
| Superior Temporal Gyrus | R | 13 | 59 | -43 | 17 | 23.90 | 0.166 | 25 |

Note: ^a^ According to the Talairach Daemon Atlas (<http://www.nitrc.org/projects/tal-daemon/>), ^b^ Based on
the Tournoux & Talairach standard brain template, ^c^ Below the ClustSim established threshold, BA= Brodmann’s Area,
*Overlapping with or proximal to a cluster reported in the main analysis

| Table S8. Brain regions demonstrating significant AUDIT, AUDIT-by-Explore, CUDIT-by-Novelty Propensity, and CUDIT-by-Novelty Propensity-by-Explore effects covarying for stimulant use | | | | | | | | |
| --- | --- | --- | --- | --- | --- | --- | --- | --- |
| Coordinates of Peak Activation^b^ | | | | | | | | |
| Region^a^ | Hemisphere | BA | x | y | z | *F* | Partial η^2^ | Voxels |
| Main Effect of AUDIT | | | | | | | | |
| Ventral Putamen/aIC/iFG* | R | 45/47/13 | 47 | 17 | 5 | 27.29 | 0.185 | 51 |
| Putamen^c^ | R | - | 29 | -19 | 2 | 18.49 | 0.133 | 11 |
| dlPFC*^c^ | R | 9/10 | 29 | 50 | 26 | 16.96 | 0.124 | 16 |
| aIC/iFG* | L | 32 | -46 | 20 | 11 | 22.06 | 0.155 | 35 |
| iFG* | R | 11 | 38 | 35 | -10 | 22.97 | 0.161 | 18 |
| dlPFC^c^ | L | 10 | -22 | 44 | 26 | 16.01 | 0.117 | 10 |
| MFG^c^ | R | 10/46 | -37 | 44 | 5 | 16.84 | 0.122 | 13 |
| Precentral Gyrus^c^ | L | 6 | -55 | 2 | 29 | 17.69 | 0.128 | 12 |
| iPL^c^ | L | 40 | -49 | -49 | 32 | 17.45 | 0.126 | 15 |
| Superior Temporal Gyrus* | R | 22 | 53 | -1 | 2 | 22.17 | 0.156 | 19 |
| Cerebellum* | L | - | -31 | -49 | -28 | 26.18 | 0.179 | 34 |
| Cerebellum*^c^ | R | - | 32 | -52 | -37 | 24.76 | 0.171 | 16 |
| AUDIT-by-Explore | | | | | | | | |
| Caudate*^c^ | L | - | -16 | 5 | 11 | 15.53 | 0.115 | 10 |
| Ventral Putamen/aIC/iFG* | R | 47 | 47 | 17 | 2 | 24.58 | 0.170 | 69 |
| dlPFC* | L | 10/46 | -37 | 44 | 5 | 17.38 | 0.127 | 21 |
| aIC/iFG*^c^ | L | 45 | -46 | 20 | 11 | 17.40 | 0.127 | 14 |
| iFG* | R | 47 | 38 | 35 | -7 | 19.14 | 0.138 | 18 |
| iPL^c^ | L | - | -49 | -52 | 35 | 16.48 | 0.120 | 10 |
| CUDIT-by-Novelty Propensity | | | | | | | | |
| iPL* | R | 40 | 47 | -40 | 38 | 20.99 | 0.149 | 40 |
| Cerebellum* | R/L | - | 5 | -76 | -28 | 19.87 | 0.142 | 20 |
| CUDIT-by-Novelty Propensity-by-Explore | | | | | | | | |
| dmPFC* | R/L | 32 | 2 | 20 | 41 | 17.92 | 0.130 | 22 |
| iPL* | R | 40 | 47 | -40 | 38 | 18.30 | 0.132 | 31 |
| Superior Temporal Gyrus | R | 13 | 59 | -43 | 17 | 25.48 | 0.175 | 25 |

Note: ^a^ According to the Talairach Daemon Atlas (<http://www.nitrc.org/projects/tal-daemon/>), ^b^ Based on
the Tournoux & Talairach standard brain template, ^c^ Below the ClustSim established threshold, BA= Brodmann’s Area,
*Overlapping with or proximal to a cluster reported in the main analysis

| Table S9. Brain regions demonstrating significant AUDIT, AUDIT-by-Explore, CUDIT-by-Novelty Propensity, and CUDIT-by-Novelty Propensity-by-Explore effects covarying for antidepressant use | | | | | | | | |
| --- | --- | --- | --- | --- | --- | --- | --- | --- |
| Coordinates of Peak Activation^b^ | | | | | | | | |
| Region^a^ | Hemisphere | BA | x | y | z | *F* | Partial η^2^ | Voxels |
| Main Effect of AUDIT | | | | | | | | |
| Ventral Putamen/aIC/iFG* | R | 45/47/13 | 53 | -1 | 2 | 31.41 | 0.207 | 107 |
| Ventral Putamen*^c^ | R | - | 26 | 14 | -1 | 18.77 | 0.134 | 10 |
| Putamen^c^ | R | - | 29 | 19 | -2 | 22.32 | 0.156 | 15 |
| dlPFC* | R | 9/10 | 29 | 50 | 26 | 17.22 | 0.125 | 24 |
| dlPFC* | L | 10/46 | -37 | 44 | 5 | 17.15 | 0.125 | 22 |
| dlPFC*^c^ | L | 9 | -22 | 44 | 26 | 18.04 | 0.130 | 13 |
| aIC/iFG* | L | 32 | -46 | 20 | 11 | 22.77 | 0.159 | 48 |
| Precentral Gyrus^c^ | L | 6 | -55 | 2 | 29 | 16.64 | 0.121 | 13 |
| iPL | L | 40 | -49 | -49 | 32 | 16.87 | 0.123 | 23 |
| Cerebellum* | L | - | -31 | -49 | -28 | 26.70 | 0.182 | 40 |
| Cerebellum*^c^ | R | - | 32 | -52 | -37 | 25.76 | 0.177 | 16 |
| AUDIT-by-Explore | | | | | | | | |
| Caudate*^c^ | L | - | -16 | 5 | 11 | 15.78 | 0.116 | 16 |
| Ventral Putamen/aIC/iFG* | R | 47 | 47 | 17 | 2 | 24.92 | 0.172 | 77 |
| aIC/iFG* | L | 45 | -46 | 20 | 11 | 18.37 | 0.133 | 22 |
| dlPFC* | L | 10/46 | -37 | 44 | 5 | 18.51 | 0.134 | 29 |
| ACC*^c^ | L | 32 | -7 | 35 | 17 | 16.30 | 0.120 | 14 |
| iFG* | R | 47 | 35 | 32 | -4 | 20.69 | 0.147 | 22 |
| iPL^c^ | L | 40 | -49 | -52 | 35 | 15.81 | 0.116 | 15 |
| CUDIT-by-Novelty Propensity | | | | | | | | |
| iPL* | R | 40 | 47 | -40 | 38 | 21.09 | 0.149 | 37 |
| Cerebellum | R/L | - | 5 | -76 | -28 | 20.89 | 0.148 | 24 |
| CUDIT-by-Novelty Propensity-by-Explore | | | | | | | | |
| dmPFC* | R/L | 32 | 2 | 20 | 41 | 18.10 | 0.131 | 21 |
| iPL* | R | 40 | 47 | -40 | 41 | 19.40 | 0.139 | 30 |
| Superior Temporal Gyrus | R | 13 | 59 | -43 | 17 | 27.82 | 0.188 | 39 |
| Cerebellum* | R | - | 8 | -79 | -25 | 19.74 | 0.141 | 23 |

Note: ^a^ According to the Talairach Daemon Atlas (<http://www.nitrc.org/projects/tal-daemon/>), ^b^ Based on
the Tournoux & Talairach standard brain template, ^c^ Below the ClustSim established threshold, BA= Brodmann’s Area,
*Overlapping with or proximal to a cluster reported in the main analysis

| Table S10. Brain regions demonstrating significant AUDIT, AUDIT-by-Explore, CUDIT-by-Novelty Propensity, and CUDIT-by-Novelty Propensity-by-Explore effects excluding current smokers | | | | | | | | |
| --- | --- | --- | --- | --- | --- | --- | --- | --- |
| Coordinates of Peak Activation^b^ | | | | | | | | |
| Region^a^ | Hemisphere | BA | x | y | z | *F* | Partial η^2^ | Voxels |
| Main Effect of AUDIT | | | | | | | | |
| Insula/iFG* | R | 45/47/13 | 56 | 23 | 5 | 26.73 | 0.201 | 76 |
| dlPFC* | R | 10 | 29 | 50 | 21 | 25.00 | 0.191 | 88 |
| dlPFC* | L | 10 | -22 | 44 | 26 | 20.12 | 0.160 | 36 |
| dlPFC* | L | 10/46 | -37 | 44 | 5 | 17.69 | 0.143 | 34 |
| dlPFC* | R | 9/10 | 29 | 50 | 26 | 22.75 | 0.114 | 18 |
| aIC/iFG* | L | 32 | -46 | 20 | 11 | 22.41 | 0.174 | 39 |
| ACC | R/L | 32 | 2 | 29 | 29 | 17.56 | 0.142 | 22 |
| Cingulate Gyrus^c^ | L | 23 | -1 | -13 | 32 | 21.64 | 0.167 | 16 |
| iFG^c^ | R | 11 | 38 | 35 | -10 | 19.70 | 0.154 | 14 |
| iPL* | L | 40 | -49 | -52 | 35 | 19.13 | 0.153 | 25 |
| Cerebellum* | L | - | -31 | -49 | -25 | 21.64 | 0.170 | 31 |
| Cerebellum* | R | - | 32 | -52 | -37 | 18.99 | 0.152 | 18 |
| Cerebellum | L | - | -28 | -61 | -28 | 16.21 | 0.133 | 18 |
| Cerebellum^c^ | L/R | - | -4 | -64 | -16 | 17.42 | 0.139 | 17 |
| Cerebellum^c^ | R | - | 44 | -52 | -28 | 20.98 | 0.163 | 14 |
| Cerebellum^c^ | L | - | -16 | -70 | -28 | 14.99 | 0.122 | 12 |
| AUDIT-by-Explore | | | | | | | | |
| Ventral Putamen/aIC/iFG* | R | 47 | 50 | 17 | 2 | 25.87 | 0.196 | 81 |
| dlPFC* | L | 10/46 | -37 | 44 | 5 | 22.68 | 0.176 | 44 |
| dlPFC* | R | 10 | 29 | 50 | 23 | 18.27 | 0.147 | 44 |
| Superior Frontal Gyrus^c^ | L | 10 | -22 | 56 | 5 | 18.46 | 0.146 | 15 |
| ACC^c^ | L | 9 | -7 | 29 | 29 | 15.84 | 0.128 | 15 |
| ACC*^c^ | L | 32 | 5 | 38 | 26 | 16.22 | 0.133 | 16 |
| dmPFC^c^ | R | 8 | 5 | 20 | 44 | 15.80 | 0.128 | 15 |
| iFG^c^ | R | 47 | 32 | 32 | -4 | 16.74 | 0.134 | 14 |
| aIC/iFG*^c^ | L | 13 | -37 | 17 | 8 | 14.35 | 0.117 | 14 |
| iPL^c^ | L | 39 | -40 | -52 | 32 | 15.36 | 0.125 | 16 |
| CUDIT-by-Novelty Propensity | | | | | | | | |
| iPL* | R | 40 | 47 | -40 | 38 | 18.88 | 0.151 | 50 |
| Precuneus | R/L | 5/7 | 2 | -37 | 47 | 15.69 | 0.129 | 23 |
| dmPFC | R/L | 32 | 2 | 17 | 41 | 16.32 | 0.133 | 18 |
| Declive | L | - | -16 | -61 | -13 | 23.97 | 0.184 | 28 |
| CUDIT-by-Novelty Propensity-by-Explore | | | | | | | | |
| dmPFC* | R/L | 32 | 2 | 17 | 41 | 16.91 | 0.138 | 22 |
| iPL* | R | 40 | 47 | -40 | 38 | 19.50 | 0.155 | 34 |
| Superior Temporal Gyrus | R | 13 | 59 | -43 | 20 | 29.11 | 0.215 | 41 |
| Precuneus | R/L | 5/7 | 2 | -40 | 47 | 15.95 | 0.131 | 19 |
| Declive | L | - | -16 | -61 | -13 | 25.03 | 0.191 | 20 |

Note: ^a^ According to the Talairach Daemon Atlas (<http://www.nitrc.org/projects/tal-daemon/>), ^b^ Based on
the Tournoux & Talairach standard brain template, ^c^ Below the ClustSim established threshold, BA= Brodmann’s Area,
*Overlapping with or proximal to a cluster reported in the main analysis

| Table S11. Brain regions demonstrating significant AUDIT and AUDIT-by-Explore interactions without including CUDIT scores in the model | | | | | | | | |
| --- | --- | --- | --- | --- | --- | --- | --- | --- |
| Coordinates of Peak Activation^b^ | | | | | | | | |
| Region^a^ | Hemisphere | BA | x | y | z | *F* | Partial η^2^ | Voxels |
| Main Effect of AUDIT | | | | | | | | |
| dlPFC* | L | 10 | -37 | 44 | 2 | 16.89 | 0.121 | 18 |
| Insula*^c^ | R | 13 | 53 | -1 | 2 | 23.07 | 0.158 | 13 |
| Cerebellum* | L | - | -31 | -49 | -28 | 16.20 | 0.169 | 25 |
| Thalamus | R | - | 14 | -22 | -1 | 26.76 | 0.179 | 24 |
| Cerebellum*^c^ | R | - | 32 | -52 | -37 | 19.71 | 0.138 | 12 |
| AUDIT-by-Explore | | | | | | | | |
| aIC/Ventral Putamen*^c^ | R | - | 26 | 14 | -1 | 18.24 | 0.129 | 11 |
| dlPFC* | L | 10 | -34 | 44 | 5 | 17.84 | 0.127 | 17 |
| Thalamus | R | - | 14 | -22 | -1 | 24.40 | 0.166 | 16 |

Note: ^a^ According to the Talairach Daemon Atlas (<http://www.nitrc.org/projects/tal-daemon/>), ^b^ Based on
the Tournoux & Talairach standard brain template, ^c^ Below the ClustSim established threshold, BA= Brodmann’s Area,
*Overlapping with or proximal to a cluster reported in the main analysis

| Table S12. Brain regions demonstrating significant CUDIT-by-Novelty Propensity and CUDIT-by-Novelty Propensity-by-Explore interactions without including AUDIT scores in the model | | | | | | | | |
| --- | --- | --- | --- | --- | --- | --- | --- | --- |
| Coordinates of Peak Activation^b^ | | | | | | | | |
| Region^a^ | Hemisphere | BA | x | y | z | *F* | Partial η^2^ | Voxels |
| CUDIT-by-Novelty Propensity | | | | | | | | |
| iPL* | R | 40 | 47 | -40 | 38 | 23.61 | 0.161 | 68 |
| iPL | R | 13 | 59 | -43 | 17 | 21.92 | 0.151 | 21 |
| iPL | R | 40 | 56 | -40 | 29 | 18.20 | 0.129 | 18 |
| Paracentral Lobule | R/L | 5 | 5 | -37 | 50 | 15.55 | 0.112 | 17 |
| Cerebellum* | L | - | -16 | -58 | -16 | 22.30 | 0.153 | 23 |
| CUDIT-by-Novelty Propensity-by-Explore | | | | | | | | |
| dmPFC*^c^ | R/L | 32 | 2 | 23 | 38 | 16.95 | 0.121 | 10 |
| iPL* | R | 40 | 47 | -40 | 38 | 17.21 | 0.123 | 19 |

Note: ^a^ According to the Talairach Daemon Atlas (<http://www.nitrc.org/projects/tal-daemon/>), ^b^ Based on
the Tournoux & Talairach standard brain template, ^c^ Below the ClustSim established threshold, BA= Brodmann’s Area,
*Overlapping with or proximal to a cluster reported in the main analysis

| Table S13. Brain regions demonstrating significant AUDIT, AUDIT-by-Explore, CUDIT-by-Novelty Propensity, and CUDIT-by-Novelty Propensity-by-Explore effects including all interactions in the model | | | | | | | | |
| --- | --- | --- | --- | --- | --- | --- | --- | --- |
| Coordinates of Peak Activation^b^ | | | | | | | | |
| Region^a^ | Hemisphere | BA | x | y | z | *F* | Partial η^2^ | Voxels |
| Main Effect of AUDIT | | | | | | | | |
| Insula*^c^ | R | 22 | 53 | -1 | 2 | 20.78 | 0.156 | 14 |
| Cerebellum* | L | - | -31 | -49 | -28 | 23.44 | 0.173 | 27 |
| AUDIT-by-Explore | | | | | | | | |
| Caudate*^c^ | L | - | -16 | 5 | 11 | 15.70 | 0.123 | 11 |
| aIC/iFG/Ventral Putamen* | R | 45 | 35 | 26 | 5 | 25.60 | 0.186 | 68 |
| iFG*^c^ | R | 45 | 47 | 29 | 2 | 17.71 | 0.137 | 13 |
| aIC*^c^ | L | 13/47 | -34 | 17 | 2 | 16.81 | 0.131 | 10 |
| ACC*^c^ | L | 32 | -7 | 35 | 17 | 13.49 | 0.100 | 14 |
| iPL^c^ | R | 40 | -43 | -52 | 35 | 14.83 | 0.109 | 14 |
| dmPFC^c^ | R | 8 | -4 | 23 | 44 | 12.39 | 0.093 | 13 |
| iFG^c^ | L | 10 | -37 | 44 | -1 | 16.06 | 0.117 | 10 |
| CUDIT-by-Novelty Propensity | | | | | | | | |
| Cerebellum | R | - | 50 | -49 | -31 | 32.97 | 0.227 | 20 |
| Cerebellum | R | - | 29 | -28 | -34 | 25.56 | 0.186 | 16 |
| CUDIT-by-Novelty Propensity-by-Explore | | | | | | | | |
| Cerebellum | R | - | 38 | -58 | -52 | 14.91 | 0.118 | 21 |

Note: ^a^ According to the Talairach Daemon Atlas (<http://www.nitrc.org/projects/tal-daemon/>), ^b^ Based on
the Tournoux & Talairach standard brain template, ^c^ Below the ClustSim established threshold, BA= Brodmann’s Area,
*Overlapping with or proximal to a cluster reported in the main analysis

| Table S14. Brain regions demonstrating significant AUDIT, AUDIT-by-Explore, CUDIT-by-Novelty Propensity, and CUDIT-by-Novelty Propensity-by-Explore effects excluding community participants with significant AUD and/or CUD symptoms | | | | | | | | |
| --- | --- | --- | --- | --- | --- | --- | --- | --- |
| Coordinates of Peak Activation^b^ | | | | | | | | |
| Region^a^ | Hemisphere | BA | x | y | z | *F* | Partial η^2^ | Voxels |
| Main Effect of AUDIT | | | | | | | | |
| aIC/iFG/Ventral Putamen* | R | 45/47/13 | 56 | 2 | 2 | 33.16 | 0.221 | 146 |
| Caudate^c^ | L | - | -13 | 8 | 11 | 14.54 | 0.111 | 12 |
| Lentiform Nucleus^c^ | R | - | 26 | -22 | -1 | 17.84 | 0.132 | 11 |
| dlPFC* | R | 10 | 29 | 50 | 23 | 18.78 | 0.138 | 36 |
| dlPFC* | L | 10/46 | -37 | 44 | 5 | 16.94 | 0.126 | 25 |
| dlPFC* | L | 10 | -22 | 44 | 26 | 17.24 | 0.128 | 19 |
| aIC/iFG* | L | 45 | -46 | 20 | 11 | 23.07 | 0.165 | 47 |
| iFG* | R | 11 | 38 | 35 | -10 | 24.80 | 0.175 | 19 |
| Precentral Gyrus^c^ | L | 6 | -55 | 2 | 29 | 18.86 | 0.139 | 11 |
| Supramarginal Gyrus | L | 40 | -49 | -52 | 35 | 15.45 | 0.117 | 22 |
| Cerebellum* | L | - | -31 | -49 | -28 | 26.71 | 0.186 | 33 |
| Cerebellum* | R | - | 32 | -52 | 37 | 25.25 | 0.178 | 17 |
| Thalamus | R | - | 14 | -22 | -1 | 24.26 | 0.172 | 18 |
| AUDIT-by-Explore | | | | | | | | |
| Caudate* | L | - | -13 | 8 | 11 | 19.18 | 0.141 | 36 |
| aIC/iFG/Ventral Putamen* | R | 45/47/13 | 35 | 23 | 2 | 32.03 | 0.215 | 149 |
| aIC/iFG* | L | 13 | -37 | 17 | 8 | 19.35 | 0.142 | 28 |
| dlPFC | L | 10/46 | -37 | 44 | 5 | 17.21 | 0.128 | 21 |
| ACC* | L | 32 | -10 | 35 | 17 | 16.24 | 0.122 | 20 |
| iPL^c^ | R | 40 | -49 | -55 | 38 | 16.33 | 0.122 | 16 |
| CUDIT-by-Novelty Propensity | | | | | | | | |
| iPL* | R | 40 | 47 | -40 | 38 | 20.68 | 0.150 | 42 |
| Cerebellum* | R/L | - | 5 | -76 | -28 | 20.74 | 0.151 | 23 |
| CUDIT-by-Novelty Propensity-by-Explore | | | | | | | | |
| dmPFC* | R/L | 32 | 2 | 20 | 41 | 17.58 | 0.131 | 21 |
| iPL* | R | 40 | 47 | -40 | 38 | 19.06 | 0.140 | 37 |
| Superior Temporal Gyrus* | R/L | 13/22 | 59 | -43 | 17 | 28.81 | 0.198 | 43 |

Note: ^a^ According to the Talairach Daemon Atlas (<http://www.nitrc.org/projects/tal-daemon/>), ^b^ Based on
the Tournoux & Talairach standard brain template, ^c^ Below the ClustSim established threshold, BA= Brodmann’s Area,
*Overlapping with or proximal to a cluster reported in the main analysis

| Table S15. Brain regions demonstrating significant AUDIT, AUDIT-by-Explore, CUDIT-by-Novelty Propensity, and CUDIT-by-Novelty Propensity-by-Explore effects controlling for age | | | | | | | | |
| --- | --- | --- | --- | --- | --- | --- | --- | --- |
| Coordinates of Peak Activation^b^ | | | | | | | | |
| Region^a^ | Hemisphere | BA | x | y | z | *F* | Partial η^2^ | Voxels |
| Main Effect of AUDIT | | | | | | | | |
| aIC/iFG/Ventral Putamen* | R | 45/47/13 | 53 | -1 | 2 | 31.55 | 0.208 | 134 |
| Putamen^c^ | R | - | 29 | -19 | 2 | 19.38 | 0.139 | 15 |
| dlPFC* | L | 10/46 | -37 | 44 | 5 | 17.89 | 0.130 | 32 |
| dlPFC* | R | 10 | 29 | 50 | 26 | 18.49 | 0.133 | 31 |
| dlPFC^c^ | L | 10 | -22 | 44 | 26 | 15.97 | 0.117 | 13 |
| aIC/iFG* | L | 45 | -46 | 20 | 11 | 22.73 | 0.159 | 52 |
| iFG* | R | 11 | 38 | 35 | -10 | 25.42 | 0.175 | 23 |
| Precentral Gyrus^c^ | L | 6 | -55 | 2 | 29 | 19.77 | 0.141 | 13 |
| Cingulate Gyrus^c^ | L | 23 | -1 | -13 | 32 | 17.74 | 0.129 | 11 |
| Supramarginal Gyrus* | L | 40 | -49 | -49 | 32 | 17.53 | 0.127 | 24 |
| Cerebellum* | L | - | -31 | -49 | -28 | 26.02 | 0.178 | 35 |
| Cerebellum* | R | - | 32 | -52 | -37 | 25.12 | 0.173 | 21 |
| AUDIT-by-Explore | | | | | | | | |
| Caudate*^c^ | L | - | -16 | 5 | 11 | 15.54 | 0.115 | 15 |
| aIC/iFG/Ventral Putamen* | R | 45/47/13 | 47 | 17 | 2 | 24.81 | 0.171 | 84 |
| aIC/iFG* | L | 45 | -46 | 20 | 11 | 17.92 | 0.130 | 22 |
| dlPFC* | L | 10/46 | -37 | 44 | 5 | 18.56 | 0.134 | 33 |
| iFG* | R | 47 | 35 | 32 | -4 | 21.63 | 0.153 | 23 |
| ACC*^c^ | L | 32 | -10 | 35 | 17 | 16.01 | 0.118 | 15 |
| iPL* | L | 40 | -49 | -52 | 35 | 16.00 | 0.118 | 17 |
| CUDIT-by-Novelty Propensity | | | | | | | | |
| iPL* | R | 40 | 47 | -40 | 38 | 20.91 | 0.148 | 38 |
| Cerebellum* | R/L | - | 5 | -76 | -28 | 19.33 | 0.139 | 19 |
| CUDIT-by-Novelty Propensity-by-Explore | | | | | | | | |
| dmPFC* | R | 32 | 2 | 20 | 41 | 17.63 | 0.128 | 21 |
| iPL* | R | 40 | 47 | -40 | 38 | 18.61 | 0.134 | 32 |
| Superior Temporal Gyrus* | R | 40 | 59 | -45 | 18 | 23.87 | 0.166 | 25 |

Note: ^a^ According to the Talairach Daemon Atlas (<http://www.nitrc.org/projects/tal-daemon/>), ^b^ Based on
the Tournoux & Talairach standard brain template, ^c^ Below the ClustSim established threshold, BA= Brodmann’s Area,
*Overlapping with or proximal to a cluster reported in the main analysis

| Table S16. Brain regions demonstrating significant effects of based on categorical analyses | | | | | | | | |
| --- | --- | --- | --- | --- | --- | --- | --- | --- |
| Coordinates of Peak Activation^b^ | | | | | | | | |
| Region^a^ | Hemisphere | BA | x | y | z | *F* | Partial η^2^ | Voxels |
| Main Effect of AUDIT Group | | | | | | | | |
| aIC/iFG/dlPFC* | L | 45 | -46 | 20 | 11 | 26.11 | 0.180 | 85 |
| aIC/iFG* | R | 45/13/47 | 38 | 23 | 5 | 26.17 | 0.180 | 55 |
| dlPFC* | R | 10 | 29 | 50 | 23 | 20.25 | 0.145 | 53 |
| dlPFC* | L | 10 | -22 | 44 | 26 | 18.48 | 0.134 | 25 |
| dlPFC^c^ | L | 10 | -40 | 50 | 5 | 16.16 | 0.118 | 12 |
| dmPFC^c^ | L | 32 | -4 | 23 | 41 | 20.99 | 0.148 | 15 |
| dmPFC^c^ | L | 8 | -13 | 35 | 38 | 15.54 | 0.114 | 12 |
| dmPFC^c^ | L | 32 | -4 | 23 | 41 | 20.99 | 0.148 | 15 |
| ACC^c^ | L | 32 | -10 | 35 | 20 | 21.21 | 0.149 | 12 |
| Insula^c^ | R | 13 | 44 | -1 | -1 | 21.55 | 0.151 | 11 |
| Precentral Gyrus | L | 6 | -55 | 2 | 26 | 24.91 | 0.173 | 22 |
| Lentiform Nucleus/Globus Pallidus/Putamen | R | - | 26 | -19 | 2 | 20.60 | 0.148 | 21 |
| iPL | L | 39 | -43 | -52 | 32 | 21.97 | 0.156 | 57 |
| Precuneus^c^ | R | 31 | 26 | -43 | 32 | 22.75 | 0.158 | 15 |
| Fusiform Gyrus | R | 20 | 41 | -37 | 16 | 20.18 | 0.145 | 19 |
| Temporal Gyrus^c^ | R | 22 | 56 | 2 | -1 | 19.47 | 0.139 | 14 |
| Temporal Gyrus^c^ | L | 22 | -52 | -37 | -1 | 15.38 | 0.113 | 11 |
| Cerebellum* | L | - | -31 | -49 | -28 | 33.00 | 0.217 | 51 |
| Cerebellum* | R | - | 32 | -52 | -37 | 26.76 | 0.184 | 26 |
| Thalamus^c^ | R | - | 8 | -25 | 8 | 17.45 | 0.126 | 12 |
| AUDIT Group-by-Novelty Propensity | | | | | | | | |
| Declive | L | 19 | -19 | -76 | -13 | 19.88 | 0.143 | 17 |
| AUDIT Group-by-Explore | | | | | | | | |
| Caudate*^c^ | L | - | -16 | 5 | 11 | 16.48 | 0.122 | 11 |
| aIC/iFG/Ventral Putamen* | R | 13/45/47 | 35 | 23 | 5 | 24.04 | 0.168 | 52 |
| aIC/iFG * | L | 13/47 | -46 | 20 | 11 | 22.29 | 0.158 | 62 |
| dlPFC*^c^ | R | 10 | 29 | 50 | 23 | 21.60 | 0.151 | 15 |
| ACC* | L | 32 | -10 | 35 | 20 | 16.23 | 0.120 | 17 |
| ACC^c^ | R | 9 | 5 | 41 | 26 | 29.87 | 0.198 | 14 |
| Precentral Gyrus^c^ | R | 9 | 59 | 5 | 26 | 18.22 | 0.131 | 11 |
| iPL* | L | 40 | -43 | -52 | 32 | 16.46 | 0.121 | 21 |
| Cerebellum^c^ | L | - | -13 | -40 | -34 | 20.73 | 0.146 | 12 |
| Cerebellum^c^ | R | - | 32 | -55 | -37 | 14.36 | 0.106 | 10 |
| Main Effect of CUDIT Group | | | | | | | | |
| Middle Temporal Gyrus | L | 39 | -46 | -58 | 23 | 20.95 | 0.150 | 25 |
| CUDIT Group-by-Explore | | | | | | | | |
| dmPFC*^c^ | L | 9 | -4 | 44 | 29 | 14.95 | 0.110 | 12 |
| iFG^c^ | L | 45 | -49 | 35 | 2 | 17.89 | 0.129 | 11 |
| iFG^c^ | L | 47 | -43 | 29 | -10 | 21.37 | 0.150 | 12 |
| Middle Temporal Gyrus | L | 39 | -46 | -58 | 23 | 20.54 | 0.147 | 27 |
| iPL*^c^ | L | 40 | -49 | -43 | 38 | 21.57 | 0.151 | 15 |

Note: ^a^ According to the Talairach Daemon Atlas (<http://www.nitrc.org/projects/tal-daemon/>), ^b^ Based on
the Tournoux & Talairach standard brain template, ^c^ Below the ClustSim established threshold, BA= Brodmann’s Area,
*Overlapping with or proximal to a cluster reported in the main analysis
